# Multiple processes acting from local to large geographical scales shape bacterial communities associated with *Phormidium* (cyanobacteria) biofilms in French and New Zealand rivers

**DOI:** 10.1038/s41598-018-32772-w

**Published:** 2018-09-26

**Authors:** Isidora Echenique-Subiabre, Anouk Zancarini, Mark W. Heath, Susanna A. Wood, Catherine Quiblier, Jean-François Humbert

**Affiliations:** 1 INRA, Sorbonne University, iEES Paris, 4 Place Jussieu, 75252 Paris, Cedex France; 2 Unité Molécules de Communication et Adaptation des Microorganismes (MCAM, UMR 7245), Muséum National d’Histoire Naturelle, CNRS, Case 39, 57 rue Cuvier, 75005 Paris, France; 3 Present Address: GEMA Center for Genomics, Ecology and Environment, Universidad Mayor. Camino La Pirámide, 5750 Santiago, Chile; 4 School of Biological Sciences, Victoria University of Wellington, PO Box 600, Wellington, New Zealand; 5 Present Address: Greater, Wellington Regional Council, Shed 39, 2 Fryatt Quay, Pipitea, Wellington, 6111 New Zealand; 6 Cawthron Institute, Private Bag 2, 7001 Nelson, New Zealand; 7 Université Paris Diderot, 5 rue T. Mann, 75013 Paris, France

## Abstract

River biofilms dominated by *Phormidium* (cyanobacteria) are receiving increased attention worldwide because of a recent expansion in their distribution and their ability to produce neurotoxins leading to animal mortalities. Limited data are available on the composition and structure of bacterial communities (BCs) associated with *Phormidium* biofilms despite the important role they potentially play in biofilm functioning. By using a high-throughput sequencing approach, we compared the BCs associated with *Phormidium* biofilms in several sampling sites of the Tarn River (France) and in eight New Zealand rivers. The structure of the BCs from both countries displayed spatial and temporal variations but were well conserved at the order level and 28% of the OTUs containing 90% of the reads were shared by these BCs. This suggests that micro-environmental conditions occurring within thick *Phormidium* biofilms strongly shape the associated BCs. A strong and significant distance-decay relationship (r_p_ = 0.7; P = 0.001) was found in BCs from New Zealand rivers but the Bray-Curtis dissimilarities between French and New Zealand BCs are in the same order of magnitude of those found between New Zealand BCs. All these findings suggest that local environmental conditions seem to have more impact on BCs than dispersal capacities of bacteria.

## Introduction

In order to better understand the causes of cyanobacterial blooms in freshwater ecosystems, numerous studies have investigated the potential relationships between heterotrophic bacteria and cyanobacteria. Based on field studies^1^ or cyanobacterial cultures^2,3^ it has been shown that bacterial communities (BCs) display dramatic changes in their structure during the development of cyanobacterial blooms. The BCs associated with the biofilm-forming benthic cyanobacteria *Phormidium* have also been shown to undergo successional changes during their development^4^. These data suggest that the development of cyanobacterial blooms may exerts strong direct and indirect influences on BCs through their modification of chemical parameters of the water (*e*.*g*., pH and oxygen concentrations) and on the availability of organic matter that is generated by their proliferation.

There is limited data available on the putative existence of spatial patterns in planktonic or benthic BCs. Studies that have investigated the paradigm “everything is everywhere, but, the environment selects” proposed by Baas-Becking and Beijerink^5^, have been performed on microbial communities with the goal of improving knowledge on the relative impact of species sorting (*i*.*e*., species are selected by environmental filtering^6^), and of dispersal limitation (*i*.*e*., micro-organisms can show dispersal limitation either if their movement to a new location is restricted or if establishment of individuals in a new location is hindered)^7^ on BC biodiversity^8–10^. These processes may lead to the differentiation of spatial patterns of biodiversity into communities. Among these processes the most studied to date is the distance-decay relationship in which similarity between biological communities decreases with increasing geographic distance^11^.

Several studies have already explored microbial communities living in stream biofilms revealing a lower diversity compared to suspended stream water communities^12,13^ and that stream biofilm communities were more influenced by environmental factors^14^ or catchment land use attributes^13^ than by dispersal limitation. Moreover, it was shown by Heino *et al*.^15^ that in microbial communities from boreal streams, local environmental conditions were the main drivers of variations; nevertheless, much of these variations remained unexplained. Recently, Peipoch *et al*.^16^ also found that BCs from epilithic biofilms in river-floodplain systems displayed spatial variations that were mainly explained by a concomitance of local and regional effects. Interestingly, a distance-decay relationship was also found between BC from epilithic biofilms, this relationship seeming to be linked to regional-scale differences in environmental conditions rather than to a real influence of the geographic distance associated to dispersal limitations.

All these studies provided interesting insights on the question of the processes driving the diversity and the spatial variations occurring in BCs living in association with photosynthetic microorganisms in biofilms but many questions still remain. Among them, the influence of the dominant photosynthetic microbial species into stream biofilms has not been taken into account. Biofilms dominated by diatoms, green algae and cyanobacteria display important differences in their characteristics (*i*.*e*., biomass, thickness) and consequently on the habitat provided to the associated BCs.

The aim of our study was to investigate, across various temporal and spatial scales, the variations occurring in the structure of BCs specifically associated with river biofilms dominated by *Phormidium* (cyanobacteria) (hereafter referred as *Phormidium* biofilms). Temporal analyses were performed by studying the BC’s changes occurring at intra seasonal to inter annual scales in the Tarn River (France). The spatial analyses were performed at scales, from the local (intra-river, Tarn River) up to the regional (inter-river in New Zealand) and global (inter-country, comparison France and New Zealand) levels. All the *Phormidium* biofilms were sampled during the summer season and the BCs were characterized using a 16S rRNA meta-barcoding approach.

## Results

### Environmental and biological parameters

**Figure 1:**
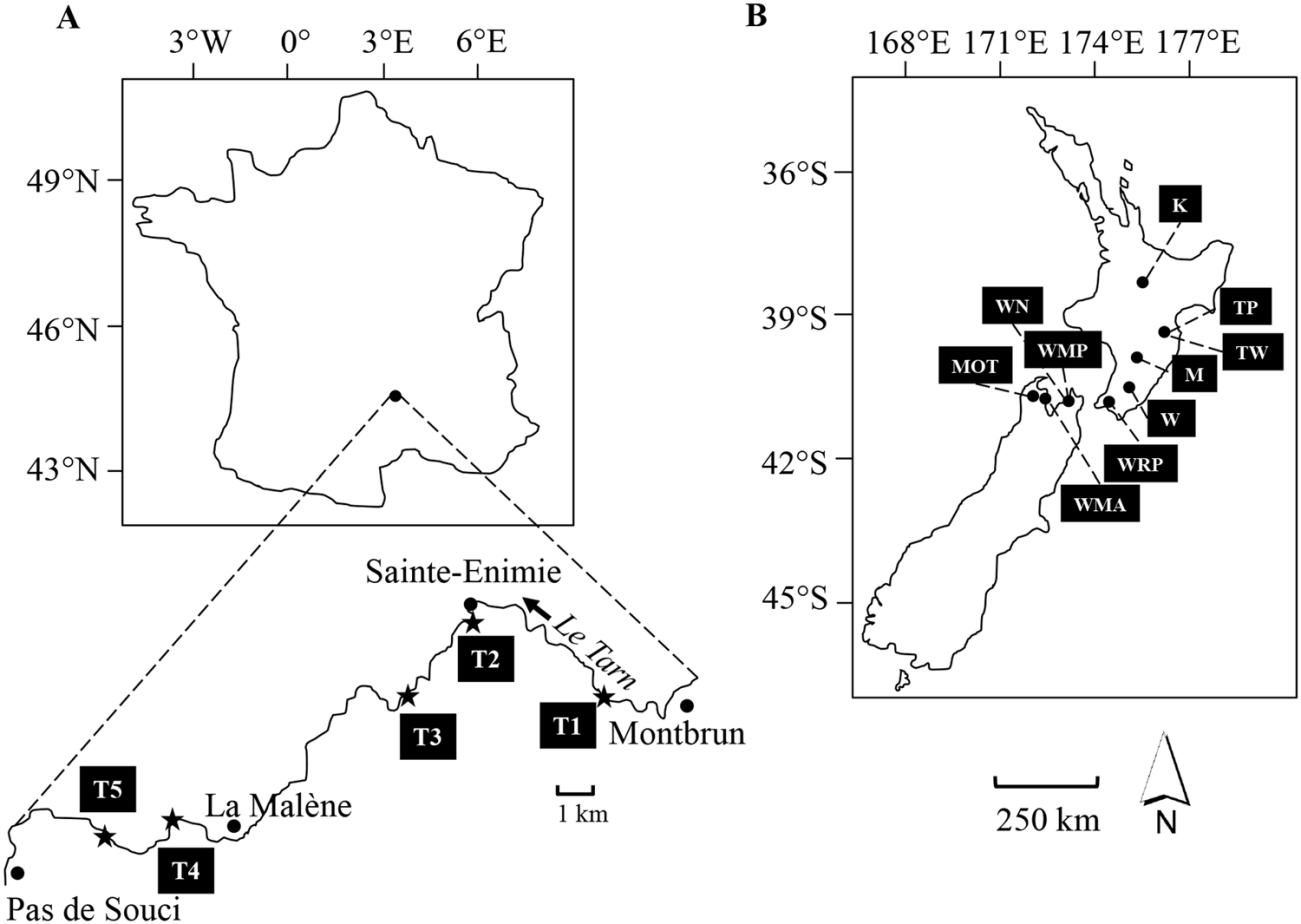
Sample sites located in France at Tarn River (**A**) sites T1: 44°20′23.08″N/3°28′2.43″E, T2 (Sainte-Enimie): 44°21′47.47″N/3°24′43.58″E, T3: 44°20′26.39″N/3°23′12.66″E, T4: 44°18′17.60″N/3°17′53.03″E and T5: 44°17′53.44″N/3°16′48.21″E) and in New Zealand (**B**) on the North Island, sites K: 38°53′19.10″S/175°46′7.54″E at Kuratau River, TP: 39°54′38.24″S/176°43′45.61″E and TW: 39°59′24.23″S/176°33′33.08″E at Tukituki River, M: 40°24′50.60″S/175°52′14.59″E at Mangatainoka River, W: 40°56′34.10″S/175°39′49.60″E at Waipoua River, WRP: 41°16′20.42″S/174°57′50.08″E at Wainuiomata River, on the South Island WN: 41°12′58.68″S/173°23′44.35″E and WMP: 41°11′13.03″S/173°25′19.24″E at Wakapuaka River, MOT: 41°15′40.25″S/172°49′16.17″E at Motueka River, WMA: 41°18′42.37″S/173°7′41.69″E at Waimea River. Countries are to scale.

Flow velocities at which biofilms were collected in the sampling sites in the Tarn River (see Fig. 1A) ranged between 0.2 ± 0.2 to 0.6 ± 0.2 m s^−1^ (lowest and highest averages by site ± SD; Supplementary Table S1). New Zealand rivers (See Fig. 1B) were within the same range (Supplementary Table S2), except for site MOT (Motueka River) where higher flow velocities were registered (*i*.*e*. 1.1 ± 0.1 m s^−1^). Overall, *Phormidium* biofilms developed at shallow depths (36 and 18.4 cm on average in the Tarn and New Zealand rivers respectively), with river water pH values close to 8.0 and temperatures above 14 °C. Biofilm biomasses (expressed in terms of Chl-*a* concentration) were very similar among the rivers, however the highest biomasses were detected in August and September (up to 30.2 ± 10.1 μg cm^−2^) in the Tarn River and in site Waipoua River (40.7 ± 8.3 μg cm^−2^) in New Zealand. Average values of *Phormidium* coverage at the bottom of the rivers were very similar (22.5 and 20.5% on average in the Tarn and New Zealand rivers respectively).

### Spatio-temporal variations in the structure of biofilm photosynthetic communities

**Figure 2:**
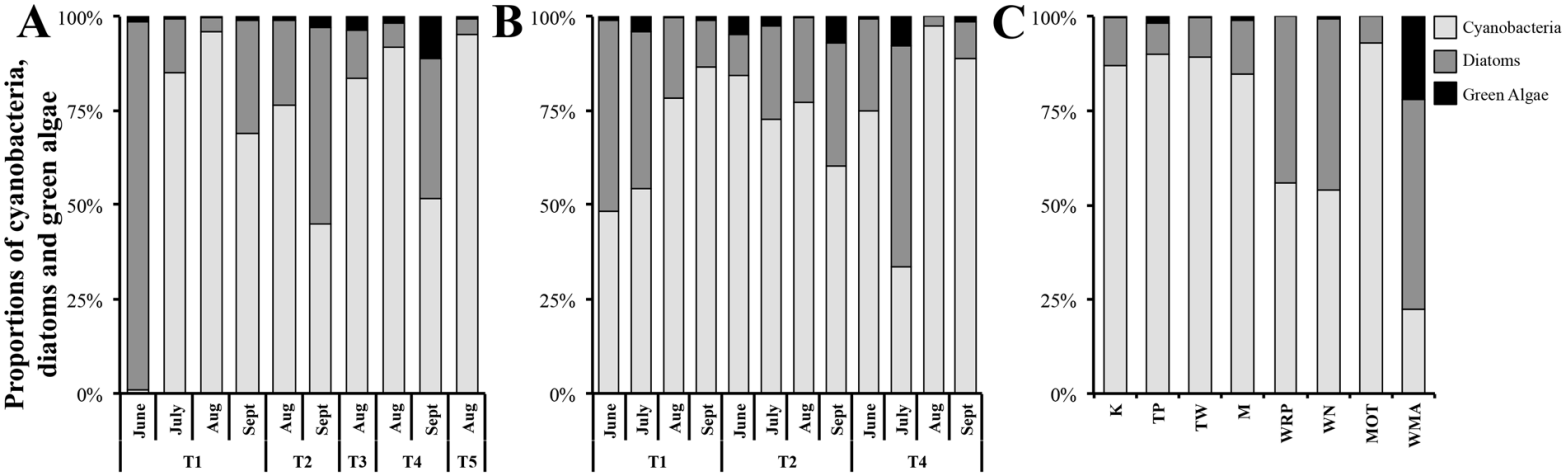
Proportions of the dominant photosynthetic microorganisms in river biofilms estimated by microscopic enumerations in Tarn River samples in 2013 (**A**) and 2014 (**B**) and in New Zealand river samples in 2013 (**C**). Stacked histograms represent average proportions of cyanobacteria = light grey, diatoms = grey, green algae = black. In (**A**) Sites T3 and T5 were only sampled in August 2013. Missing data points in (**A**) sampling months: June, July in T2 and T4 are due to (i) high flow velocities caused by high rain events, which did not allow us to make the sampling without risk for the operators or/and (ii) the lack of *Phormidium* biofilm at the site during the sampling campaign. In (**C**) sites W and WMP are not presented due to missing data (refer to Fig. 1 for site names and locations).

Microscopic analysis demonstrated that the biofilms were dominated by cyanobacteria (79.2% ± 19.2; predominantly *Phormidium* sp. Tarn 2013: 97.4% ± 13.0; Tarn 2014: 99.8% ± 0.7; New Zealand 2013: 94.6% ± 20.5), followed by diatoms (19.0% ± 18.1) and green algae (1.8% ± 3.6) (Fig. 2). This pattern was observed in all samples except; (i) June site T1 in 2013 and 2014 (see Fig. 1 for site names and locations), (ii) September site T2 in 2013, (iii) July site T4 in 2014, and (iv) WMA in New Zealand, where diatoms were dominant (63.7% ± 31.4).

In the Tarn River, the proportion of cyanobacteria varied mostly according to the sampling date (Fig. 2A,B; Supplementary Table S3). The lowest cyanobacterial proportions were observed in June 2013 and July 2014 (Fig. 2A,B) shortly after two large flow events (Supplementary Fig. S1), and the highest were observed in August and September.

In New Zealand rivers, significant differences were observed in cyanobacterial proportions between site WMA and the rest of the sampling sites (ANOVA test, all *P* < 0.004; Fig. 2C and Supplementary Table S3).

### Spatio-temporal variations in bacterial community structure at high taxonomic ranks

Analysis of the 16S rRNA gene sequences showed that biofilms were dominated by cyanobacteria (mean 84.3% ± 3.5 of the total reads per sampling campaign), mainly belonging to Oscillatoriales order (Supplementary Fig. S2) and to *Phormidium* (mean 98.5% ± 0.9 of the cyanobacteria reads per sampling campaign). We found the same 16S rRNA dominant *Phormidium* OTU (with a 97% cut-off) in all rivers (France and New Zealand), which displayed a 100% sequence similarity (Basic Local Alignment Search Tool, BLAST analysis on GenBank^TM^, KF770970) with *Phormidium uncinatum*. Excluding cyanobacteria and no-hit (CNH), the most abundant bacterial phyla were Proteobacteria (mean 69.4% ± 0.9) and Bacteroidetes (mean 23.6% ± 1.2) (Supplementary Fig. S2). Within Proteobacteria, Alphaproteobacteria (mean 26.4% ± 4.4) and Betaproteobacteria (mean 21.3% ± 1.6) and to a lesser extent Gammaproteobacteria (mean 9.0% ± 0.8), were most prominent. Unclassified Proteobacteria also accounted for a high proportion (mean 12.0% ± 4.0) of the reads in this phylum. Within these three classes, most of the sequences were classified in the orders of Rhodobacterales/Sphingomonadales, Burkholderiales and Xanthomonadales, respectively. Bacteroidetes were mainly represented by Flavobacteriales (Tarn River) and Sphingobacteriales (New Zealand rivers; Supplementary Fig. S2 and Table S4). At these high taxonomic ranks, the bacterial structure in biofilms displayed a strong similarity during the two sampling years in Tarn River (Supplementary Fig. S2; Bray-Curtis dissimilarity = 0.18 in Supplementary Table S5) and when comparing Tarn River and New Zealand rivers BC (Bray-Curtis dissimilarity = 0.20 in Supplementary Table S5).

**Figure 3:**
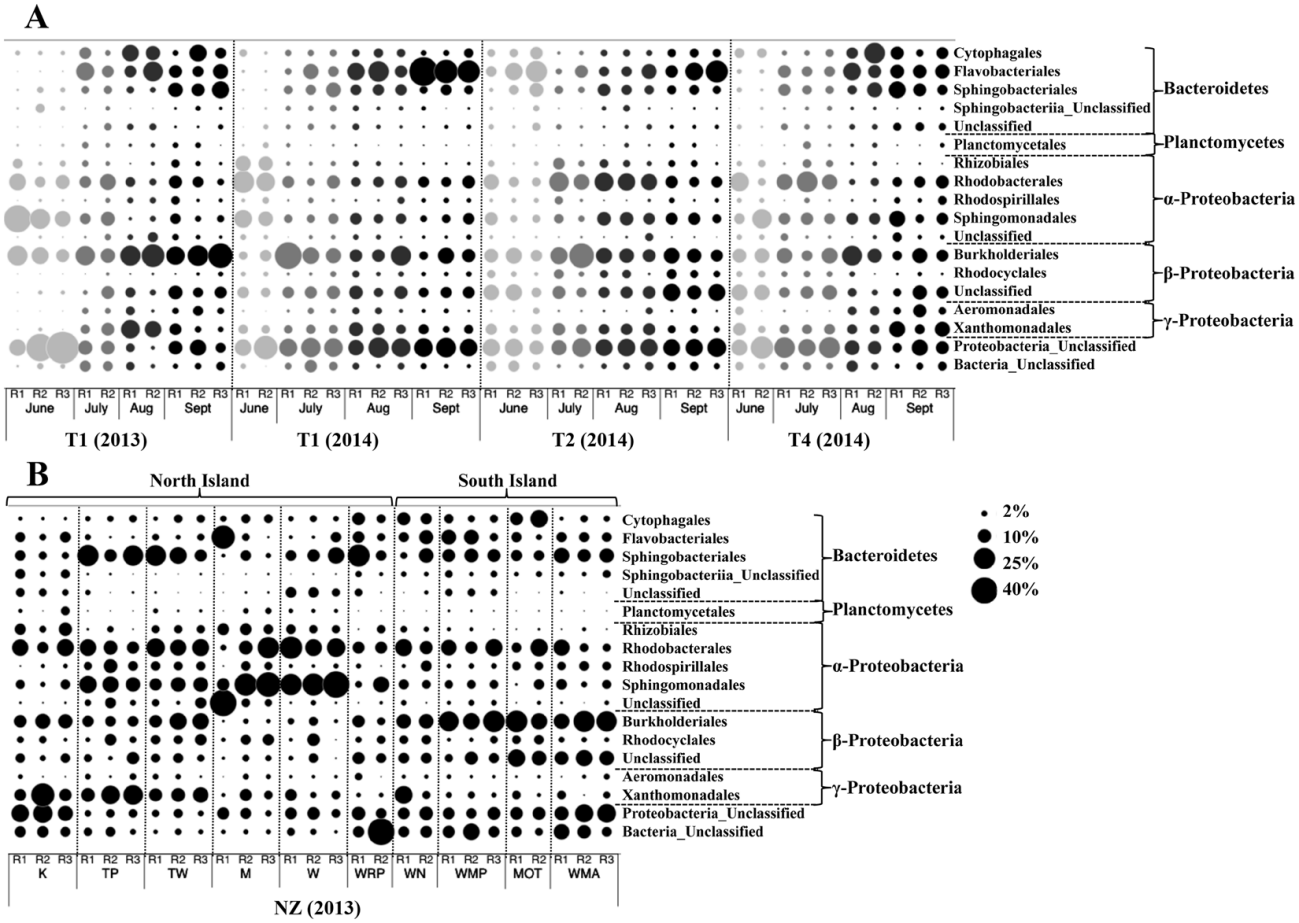
Spatio-temporal variation of the most abundant (≥1%) bacterial orders (excluding cyanobacteria and no-hit) associated with *Phormidium* biofilms: (**A**) Tarn River at site T1 in 2013 and T1, T2 and T4 in 2014 from June (light grey) to September (black). In 2013 at the Tarn River, only samples from site T1 were selected and showed here as having complete temporal analysis; (**B**) New Zealand (NZ) rivers (refer to Fig. 1 for site names and locations). Circles represent proportion. R1, R2 and R3 correspond to replicates. Abbreviations: Aug refer to August and Sept to September.

**Figure 4:**
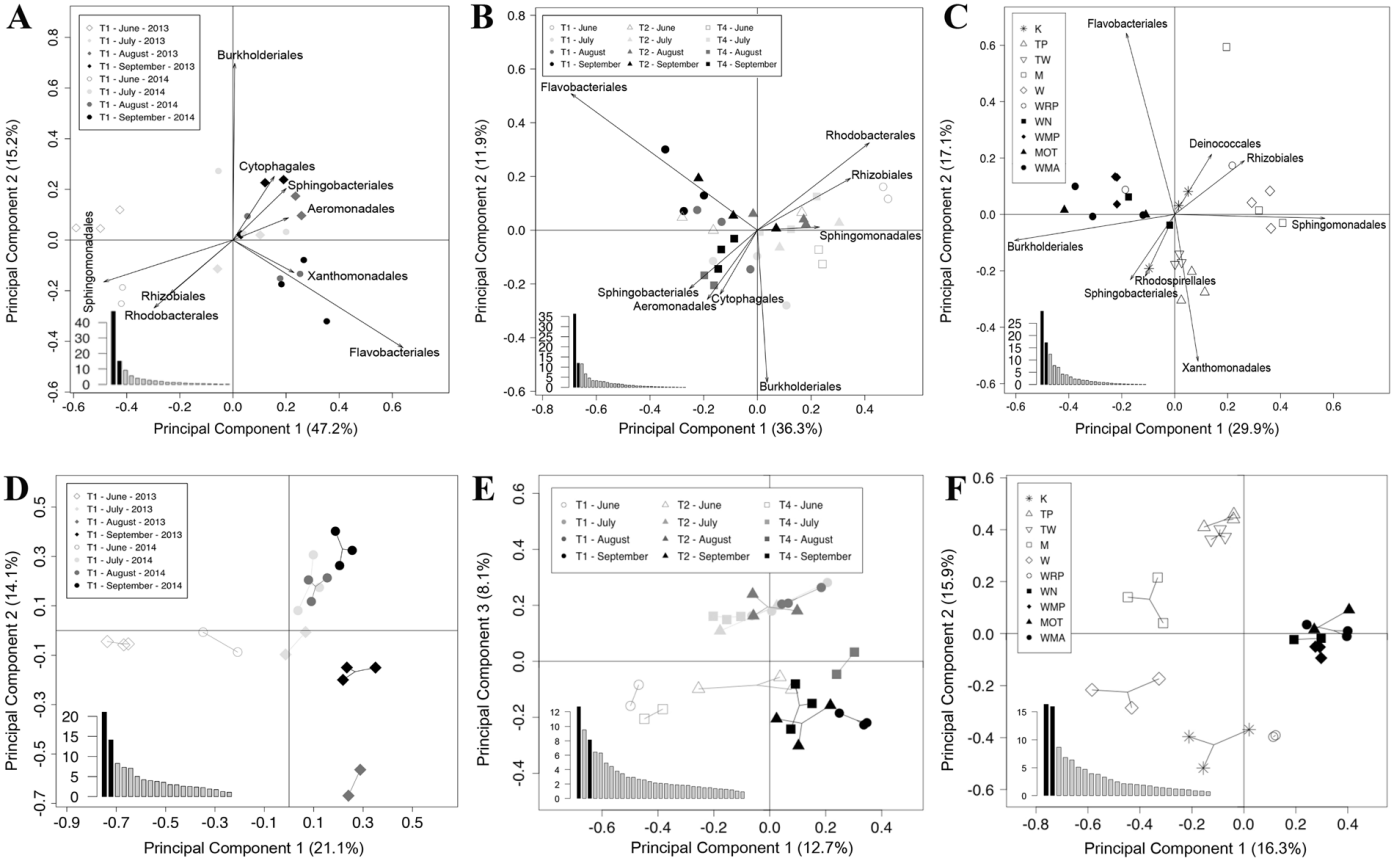
Principal Component Analysis (PCA) of bacterial orders (most abundant; ≥1%) (**A**–**C**) and operational taxonomic unit (OTU) (**D**–**F**). Relative abundances based on 16S rRNA gene fragment sequences (excluding cyanobacteria and no-hit sequences) of biofilm samples from: Site T1 from Tarn River in 2013–2014 (**A**,**D**), Tarn River in 2014 (**B**,**E**), and New Zealand rivers (**C**,**F**) (refer to Fig. 1 for site names and locations). Histograms on the bottom-left part of the graph represent the percentages of variation explained by each principal component. The two components selected for the two-dimensional representation are highlighted in black.

When comparing the occurrences and relative abundances of dominant bacterial orders (≥1%; excluding CNH) in Tarn and New Zealand rivers, it appeared that the global structure of all the BC was maintained among sampling sites (see Fig. 3). However, when performing Principal Component Analyses (PCA) on three different data sets (Tarn 2013 & 2014 Site T1, Tarn 2014 and New Zealand rivers), spatio-temporal variations were evident in the structure of the BCs. The PCA performed on the biofilms collected in T1 sampling station from the Tarn River (Fig. 4A), highlighted on the first axis (47.2% of the total inertia) of the projection, a highly significant distinction (PERMANOVA test, *P* = 0.001; Table 1) between the samples collected in June and those collected from July to September. These seasonal differences in the structure of the BCs were mainly due to the relative importance of Sphingomonadales, Rhizobiales and Rhodobacteriales orders (ANOVA test, all *P* < 0.001; Supplementary Table S6). On the second axis of this PCA (15.2% of the total inertia; Fig. 4A), a significant difference (PERMANOVA test, *P* = 0.002; Table 1) was found between samples collected in 2013 and 2014. In concordance with sampling month differentiation observed in site T1, when considering all sampling sites from Tarn 2014 (Fig. 4B), the same temporal pattern was obtained (first axis explaining 36.3% of the total inertia; PERMANOVA test, *P* = 0.001; Table 1). The PCA performed on the New Zealand rivers highlighted significant differences (Nested PERMANOVA test, *P* = 0.02; Table 1) between BCs from the North and the South Islands of New Zealand. These differences were mainly due to the relative abundances of bacteria belonging to Burkholderiales and Sphingomonadales orders (Fig. 4C). Finally, significant differences (Nested PERMANOVA test, *P* = 0.003; Table 1) were found also at the order level, between BCs from New Zealand rivers and the Tarn River (see PCA Supplementary Fig. S3A).

**Table 1:** PERMANOVA results within and between sampling campaigns excluding cyanobacteria and no-hits (CNH) at the order and operational taxonomic unit (OTU) levels.

Sampling campaigns	Source	df	Order level			OTU level		
			MS	Pseudo-F	*P*	MS	Pseudo-F	*P*
Site T1 2013–2014	Month	3	0.166	6.32	**0.001**	0.440	3.71	**0.001**
	Year	1	0.111	4.24	**0.002**	0.506	4.27	**0.001**
	Month*Year	3	0.055	2.09	**0.018**	0.289	2.44	**0.001**
	Residual	13	0.026			0.119		
	Total	20						
Tarn 2014	Site	2	0.054	2.26	**0.005**	0.261	2.28	**0.001**
	Month	3	0.086	3.60	**0.005**	0.340	2.96	**0.001**
	Site*Month	6	0.056	2.37	**0.001**	0.207	1.80	**0.001**
	Residual	20	0.024			0.115		
	Total	31						
Tarn 2013–2014	Site	4	0.057	2.16	**0.004**	0.232	1.95	**0.001**
	Month	3	0.132	5.02	**0.001**	0.443	3.72	**0.001**
	Year	1	0.124	4.72	**0.001**	0.557	4.67	**0.001**
	Site*Month	6	0.064	2.45	**0.001**	0.234	1.97	**0.001**
	Site*Year	2	0.072	2.73	**0.001**	0.248	2.08	**0.001**
	Month*Year	3	0.059	2.23	**0.002**	0.297	2.49	**0.001**
	Site*Date*Year	2	0.035	1.32	0.153	0.174	1.46	**0.024**
	Residual	33	0.026			0.119		
	Total	54						
New Zealand	Island	1	0.229	2.68	**0.021**	0.721	2.01	**0.042**
	Site(Island)	8	0.085	3.00	**0.001**	0.359	3.17	**0.001**
	Residual	17	0.028	0.03		0.113	0.11	
	Total	26						
Tarn & New Zealand	Country	1	0.247	2.82	**0.001**	1.337	3.84	**0.001**
	Site(Country)	13	0.088	2.22	**0.001**	0.348	2.14	**0.001**
	Residual	67	0.039	0.04		0.162	0.16	
	Total	81						

A total of 999 permutations were performed. Site(Country) or Site(Island) means that the term on the left is nested inside the term in brackets. Asterisk denotes interaction between factors. df: degrees of freedom, MS: means of squares. Bold values: *P* < 0.05.

### Spatio-temporal variations in bacterial community structure at the OTU level

Rarefaction curves constructed from the dataset including all the sequences (at least 8,918 sequences per sample) were close being asymptotic for most of them (see Supplementary Fig. S4A–C). After removing CNH sequences (remaining sequence number of at least 402 sequences per sample), the rarefaction curves were not asymptotic. Consequently, further analyses were performed only on the dominant OTUs from the BCs (see Supplementary Fig. S4 D–F).

When all the sequences were taken into account, 534 OTU (24.3%) of the 2,198 OTU were shared by samples collected in Tarn River in 2013 and 2014 and in New Zealand rivers in 2013 (Supplementary Fig. S5A). Similar results were obtained when excluding CNH sequences (283 shared OTU, representing 28.2% of total OTU; Supplementary Fig. S5B). Shared OTUs (=core OTUs) contained a very high number of reads compared to those only present in biofilms from Tarn or New Zealand rivers (Supplementary Fig. S5) and there was a clear relationship between the mean number of reads per OTU and the number of samples in which each OTU was found (see Supplementary Fig. S6). At the genus level, the OTUs belonging to the core species (≥1% of total reads) were represented mainly by *Rhodobacter*, *Pedobacter*, *Silanimonas*, *Flavobacterium*, *Hydrogenophaga*, *Runella*, *Tolumonas* and *Sphingobacterium* (50 most abundant OTUs are presented in Supplementary Table S7).

In agreement with the analysis on BCs at the order level, PCA performed at the OTU level reveal the same spatio-temporal variations in BCs structure (Fig. 4D–F). Significant temporal differences (PERMANOVA test, all *P* = 0.001; Table 1) in BCs were found when comparing samples collected in the Tarn River at site T1 between June and the other months (Fig. 4D; first axis explaining 21.1% of the total inertia) and between the two sampling years (second axis explaining 14.1% of the total inertia). The PCA performed on the BC sampled in 2014 in the Tarn River (Fig. 4E) confirmed the existence of temporal variations with a distinction among samples from June, July-August and September and showed also spatial variations among the different sites (PERMANOVA test, all *P* = 0.001; Table 1). The structures of BCs associated with *Phormidium* biofilms from New Zealand rivers located in the North Island display significant differences with those of rivers located in the South Island (Fig. 4F; Nested PERMANOVA test, *P* = 0.04; Table 1). In addition, BCs from South Island biofilms were tightly grouped (Fig. 4F; Bray-Curtis dissimilarity = 0.45 in Supplementary Table S5), whereas those from the North Island were much more dispersed (Bray-Curtis dissimilarity = 0.61 in Supplementary Table S5). Moreover, biofilms from the North Island followed a gradient of ordination from north to south on the second axis of the analysis (Fig. 4F), except for site K. Finally, significant differences (Nested PERMANOVA test, *P* = 0.001; Table 1) were found at the OTU level, between BC from New Zealand rivers and those from the Tarn River (see PCA Supplementary Fig. S3B).

**Figure 5:**
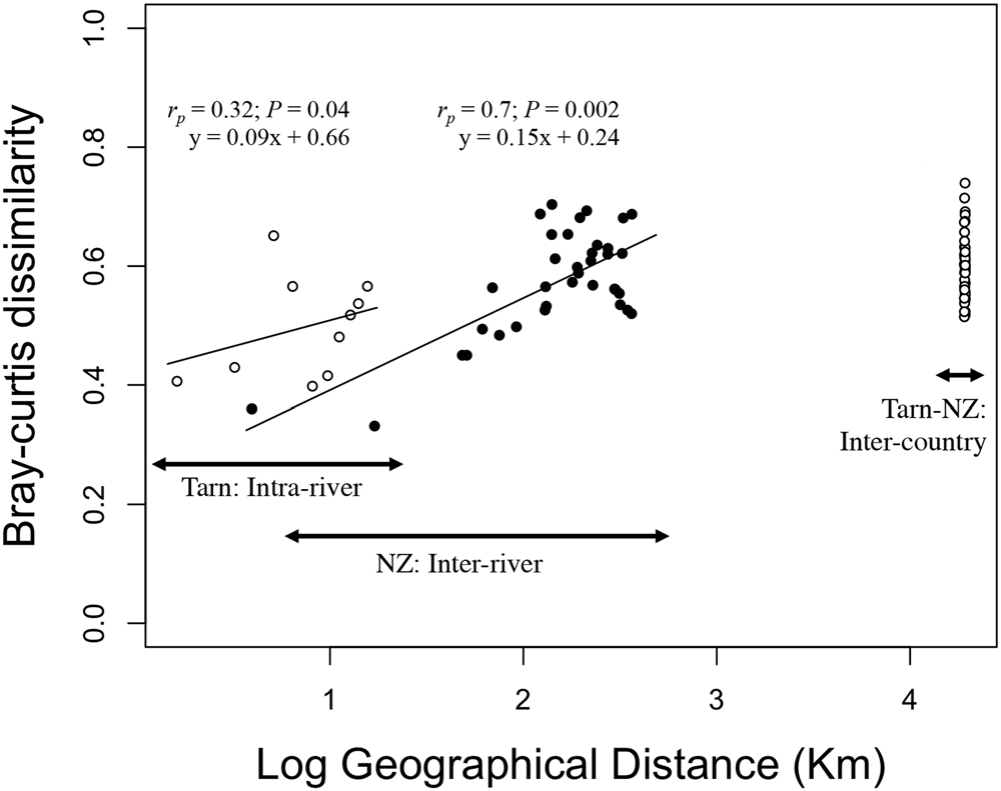
Geographic distance and Bray-Curtis dissimilarity relationship in the Tarn and New Zealand rivers. Bray-Curtis dissimilarities were performed on operational taxonomic units (OTUs) based on 16S rRNA relative abundances of pooled replicates excluding cyanobacteria and no-hit. Comparisons were conducted among Tarn River biofilms harvested in August 2013 (open circles on the left), biofilms from New Zealand (NZ) rivers (black circles in the middle) and between Tarn and New Zealand river biofilms (open circles at the right). Mantel statistics are based on Pearson’s product-moment correlation.

There was a weak significant relationship (Mantel test, r_p_ = 0.32; *P* = 0.04; Fig. 5) between Bray-Curtis dissimilarities and geographical distances among the BCs collected in five sampling sites of the Tarn River in August 2013 (Fig. 5). Conversely, this relationship was highly significant among BCs sampled in New Zealand rivers (Mantel test, r_p_ = 0.73; *P* = 0.001; Fig. 5). Finally, while the geographical distance between France and New Zealand is much greater that the geographical distances between New Zealand rivers, the Bray-Curtis dissimilarity values estimated between BCs from Tarn and New Zealand rivers are in the same order of magnitude than those found between the most distant rivers in New Zealand (Fig. 5; Average Bray-Curtis dissimilarity values presented in Supplementary Table S5). A similar curve was found after transforming abundance data to presence/absence data using Jaccard index of dissimilarity (Supplementary Fig. S7).

## Discussion

Numerous studies in the twenty past years have highlighted the major role microbial biofilm communities play in stream ecosystem functioning (see the review of Battin *et al*.)^17^. In particular, stream biofilms contain an assemblage of phototrophic and heterotrophic microbial communities, which contribute to primary production and numerous biogeochemical cycles. The relationships between phototrophic and heterotrophic taxa can be positive and negative, and these interactions may also be important in shaping biofilm communities^17,18^. In addition, it is well known that environmental factors can have direct and indirect influences on the microbial communities of stream biofilms^19–21^.

In this study, we investigated stream biofilms whose phototrophic component was dominated by cyanobacteria belonging to the *Phormidium* genus for three reasons. These cyanobacteria are potentially toxic and their proliferations seems to be increasing worldwide^22,23^. Secondly, biofilms dominated by *Phormidium* are found in similar environmental conditions in streams (*i*.*e*., depth, flow velocity, substrate)^24–26^. This reduces the number of potential environmental factors that might impact BCs associated with *Phormidium* and enabled a comparative approach to be performed on these communities between France and New Zealand rivers. Finally, biofilms dominated by *Phormidium* are characterized by a large thickness (up to several millimetres), which results in specific micro-environmental conditions and cyanobacteria probably constitutes the main source of organic compounds for the BC. Consequently, we anticipated that: (i) BCs associated with *Phormidium* would display a conserved structure at various spatiotemporal scales, and (ii) that the BCs differ from those living in biofilms dominated by other phototrophic microorganisms, for example diatoms, in the same kind of environments.

In agreement with our hypothesis that the specific micro-environmental conditions provided by thick *Phormidium* biofilms shape the BCs structure, we showed that all the BCs associated with *Phormidium* biofilms in the Tarn River and New Zealand rivers display similar patterns in their structure. At higher taxonomic ranks (phylum, class or order), these BCs were dominated by species belonging to Proteobacteria (Alpha- and Betaproteobacteria) and Bacteroidetes phyla, the three most abundant genera being *Rhodobacter* (Alphaproteobacteria), *Flavobacterium* (Flavobacteria) and *Pedobacter* (Sphingobacteria). The dominance of Proteobacteria and Bacteroidetes is a common feature of BCs in biofilms of streams^12,21,27^ and lakes^28^. However, when analysing the few studies available using NGS approaches on BCs from river biofilms, it appears that these BCs are dominated by various genera, including *Acidovorax* (Betaproteobacteria), *Flavobacterium* and *Polynucleobacter* (Betaproteobacteria) in the study of Besemer *et al*.^12^; *Arenimonas* (Gammaproteobacteria), *Rhodobacter* and Betaproteobacteria-undefined genus in the study of Bricheux *et al*.^29^ and *Rhodobacter*, *Exiguobacterium* (Firmicutes) and *Zymomonas* (Alpha) in the study of Peipoch *et al*.^16^.

Furthermore, our data indicates that habitat type (benthic *versus* pelagic life) is a key factor acting on BCs associated with cyanobacteria. The importance of habitat on BCs associated with *Phormidium* biofilms in rivers is further supported by the fact that these communities display marked differences with those associated with planktonic bloom-forming cyanobacteria in lakes, which are commonly dominated by Betaproteobacteria^1,30^ (*i*.*e*., Comamonadaceae members)^30^ or Bacteroidetes^31,32^ (*i*.*e*., *Flavobacterium*, *Sediminibacterium*^31^), and in a lesser extent by Alpha- (*i*.*e*., *Pelagibacter*)^31^ and Gammaproteobacteria^1,31^ (i.e., *Pseudomonas*)^31^.

When comparing BC in biofilms dominated by diatoms and by cyanobacteria in Tarn River, marked differences were found between them at all taxonomic levels. The BCs associated with diatoms in biofilms were largely dominated by Alphaproteobacteria as previously observed in marine environments^33^, in epilithic lake biofilms^34^, and in river biofilms^18^. These findings suggest that BC are at least to some extent dependent on the key phototroph microorganisms in the biofilms, and the resulting micro-environmental conditions. Among the processes that could be involved in the selection of bacterial species inside the biofilms, Becker *et al*.^35^ showed that the dissolved organic matter (DOM) produced by diatoms and cyanobacteria is very different, which potentially has a substantial influence on heterotrophic communities utilising it for their growth. Wagner *et al*.^36^ also found that the quality of the DOM impacted the functional and structural diversity of BCs in hyporheic biofilms. *Phormidium* biofilms can be several millimetres thick and form very cohesive biofilms^23,37^. A recent study has shown that this creates conditions within the biofilm (*e*.*g*., pH, dissolved oxygen, nutrient and metal concentrations) that are very different from those of the overlying water^37^. It is likely that these micro-environmental conditions have a substantial impact on the BC structure, which could partly explain the dominance of particular OTUs into the BCs, regardless of their geographical origin (France or New Zealand). As *Phormidium* biofilms grow, these within-biofilm conditions probably become more intense and this may explain why marked shifts in BCs were observed during different successional phases^4^.

We identified a positive relationship between the abundance of reads per OTU and the number of samples in which they were retrieved. This indicates that the most abundant OTUs are widely distributed and constitute the core species of the BC associated with *Phormidium* biofilms in rivers. A similar positive relationship has been previously described by Humbert *et al*.^38^ for pelagic BCs from lakes and by Dougal *et al*.^39^ for BCs in the large intestine of horses. As members of the core species, it is probable that these OTUs play a major role in the basic functioning of biofilm BCs while satellite species distributed in a restricted number of samples, are probably involved in the adaptation to local environmental conditions. Many of the dominant OTUs in the biofilms examined in this study are well known for their ability to degrade complex compounds such as recalcitrant humic substances or organic contaminants (*e*.*g*., *Sphingomonas*^40^ and *Hydrogenophaga*^41^) or large organic polymeric proteins and polysaccharides (*e*.*g*., *Flavobacterium*^42^ and *Runella*^43^). Others have been shown to be involved in the nitrogen cycle, including nitrogen fixation and denitrification (*e*.*g*., OTUs from *Rhodobacter*^44^, *Sphingomonas*^40^, *Azonexus*^45^, and *Hydrogenophaga*^41^). During the early stages of development of the *Phormidium* biofilms (June and July in Tarn River), numerous reads belonging to *Rhodobacter* genus were identified. This genus is metabolically diverse, with members that perform anaerobic phototrophy and photoautotrophy to aerobic chemoheterotrophy^44^. The high abundance of these purple bacteria including OTUs belonging to Burkholderiales, Rhizobiales, Rhodocyclales, Rhodospirillales and Rhodobacterales raises questions regarding their potential contribution to primary production in river biofilms^18^.

Distance-decay similarities allow variations in beta-diversity across different spatial scales (*e*.*g*. Soininen *et al*.^46^; Tan *et al*.^47^) to be explored. Beta-diversity depends on the sampling scale and should increase with increasing spatial extent of the study area^47^. In our study, three different scales were taken into account, from local (intra-river), to regional (inter-river) and global (inter-country) levels. Different processes drive beta-diversity at these different spatial scales. For example, topology, altitude, discontinuous habitat, latitudinal gradients in productivity, and climate act as regional scales, while habitat structure and composition act as local filters^48^.

As expected, the distance-decay relationship estimated between BCs associated with *Phormidium* biofilms collected in the five sampling stations of the Tarn River (intra-river comparison) was much lower than that found among BCs collected in various rivers in New Zealand (inter-river comparison). However, it was unexpected to find that the genetic dissimilarities between BCs from France and New Zealand were of the same order of magnitude as those found between the most distant rivers in New Zealand. We had anticipated that the larger distance between France and New Zealand would limit the dispersion of bacteria, and that other processes such as climate factors and continental isolation which act as large-scale filters^48^, would result in much greater differences among these communities. This result is largely due to the presence of several core OTUs, which are highly abundant in *Phormidium* biofilms from both France and New Zealand.

Collectively these results indicate that in addition to micro-environmental conditions that shape the BCs associated with *Phormidium* biofilms, the main processes acting on these BCs seems to be mainly related to local environmental conditions rather than to geographic distance *per se*. This is supported by the study of Lear *et al*.^13^ performed across New Zealand where they found that stream biofilm BCs were more influenced by catchment land use attributes than by dispersal limitation and also by Peipoch et *et al*.^16^ in USA.

Our data are also interesting to consider in the context of the meta-analysis on stream microbial communities provided by Zeglin^20^ and on the meta-community concept, which can be defined as a set of local communities linked by dispersal of multiple interacting species^49^. According to Zeglin^20^, BCs associated with *Phormidium* biofilms seem to be largely structured by local heterogeneity and by temporal variations, while longitudinal (from upstream to downstream) differences were less significant. The meta-community paradigms as described by Leibold *et al*.^49^ (*i*.*e*., patch-dynamic, species-sorting, mass-effect and neutral perspectives) allow us to consider our results in a theoretical framework. The development of thick *Phormidium* biofilms creates well-defined local environmental conditions (*e*.*g*., habitat, chemical gradients, organic matter availability) that may delimit ecological niches leading, by a species-sorting process, to the selection of BCs displaying a similar structure. Inside these biofilms, the local dynamics of species due to stochastic and/or deterministic processes (including seasonal succession) seems to have a marked effect on the BCs associated with *Phormidium* biofilms as emphasized by the importance of intra-site variations.

In conclusion, our findings obtained on the BCs associated with *Phormidium* biofilms collected in France and New Zealand have shown that various processes functioning at different temporal and spatial scales, shape these BCs. Among these processes, we found a distance-decay relationship between BCs collected in rivers from NewZealand. Future research should focus on a large scale sampling including other countries. Moreover, this sampling should also take into account the influence of temporal variations in the structure of BCs associated with *Phormidium* biofilms by targeting them at different phases of their development. Finally, a significant challenge for the future will be also to establish to what extent the recent increase in benthic *Phormidium* proliferations in river worldwide is due to perturbations of heterotrophic BCs in biofilms, or whether it is a result of changes in environmental factors and processes promoting the development of *Phormidium*, with subsequent consequences on the BCs.

## Methods

### Sites description

Samples were collected from nine wadeable rivers in France and New Zealand. Previous studies had identified the presence of potentially toxic benthic cyanobacteria in these rivers^50–52^. In France, samples were collected at five sites (T1 to T5; see Fig. 1A) in the Tarn River between June and September 2013 and 2014, which is the most favourable period for the growth of *Phormidium* biofilms. Sites T3 and T5 were only sampled in August 2013 as part of an expanded monitoring campaign. In New Zealand, ten sites located in eight rivers were sampled in February 2013 (see Fig. 1B). The bed substrates at all sites in France and New Zealand were dominated by cobble and boulder (6 to 26 cm length), except at the Kuratau River in New Zealand (site K; Fig. 1B), where sand was the most abundant substrate. Nitrate (NO_3_-N) and total phosphorus (TP) concentrations were higher at the Tarn River (mean 1.4 ± 0.6 mg L^−1^ and 0.02 ± 0.01 mg L^−1^ respectively; data extracted from the French database SIE Adour-Garonne^53^); compared to values from New Zealand rivers (mean 0.4 ± 0.4 mg L^−1^ and 0.01 ± 0.01 mg L^−1^ respectively; mean values collected at each sampling site according to previously described^4^). At each sampling point, water temperature, pH, flow velocity and depth were measured. In The Tarn River, variations in flow rates during the sampling period at two survey stations (Bedoues located 28 km upstream, and Mostuéjouls located 38 km downstream from Sainte-Enimie) were obtained from hydro France database^54^. Sites TP and TW were both located in the Tukituki River (North Island) and sites WN and WMP in the Wakapuaka River (South Island; Fig. 1B). Rivers are very dynamic systems and thus biofilms developing in these ecosystems are subjected to changes occurring in water flow/velocities. Missing data points in Fig. 1 are a consequence of (i) high flow velocities caused by high rain events, which did not allow us to make the sampling without risk for the operators or/and (ii) the lack of *Phormidium* biofilm in the site during the sampling campaign.

### Sample collection

For all sampling sites and dates, except for site T1 in June 2013, a grid (10 m × 10 m) was set up in riffle habitat (shallow region of river with fast flow, mainly over 0.3 m s^−1^). Sampling was performed on ten points selected randomly inside the grid using a random number table. At each sampling point delimited by a single field of view of an underwater viewer (707 cm^2^), the percentage of coverage of the river substrate colonized by *Phormidium* biofilms was visually estimated (independently by two operators). *Phormidium* cover (%) for a single site was consecutively calculated by averaging the estimated values from the 10 sampling points. A single cobble with a *Phormidium* biofilm was sampled. Biofilms were removed using sterile tweezers. Subsamples were taken for DNA and chlorophyll-*a* (Chl-*a*) extraction, and a known area (1 cm^2^) for later microscopic identification and enumeration (preserved immediately with Lugol’s iodine solution). Samples were stored chilled in the dark, and subsequently frozen (−20 °C), except for the samples for microscope analysis which were stored at 4 °C.

For site T1 in June 2013, where *Phormidium* development was low (percentage coverage < 5% of the river substrate) and biofilms were thinner, sampling was undertaken at three points along a transect parallel to the water’s edge and positioned at two meters from the shoreline. At each point, all cobbles (5 to 20 cm length) visible in a single field of view of the underwater viewer were collected. The cobbles were scrubbed and the biomass collected in 150 mL of river water. Aliquots (5 mL) were filtered for Chl-*a* (GF/C Whatman) and DNA extraction (Polycarbonate 0.2 μm GTTP Millipore). Samples were stored chilled in the dark, and subsequently frozen (−20 °C) for later analysis in the laboratory. Subsamples (1 mL) were fixed with Lugol’s iodine solution and stored at 4 °C for later microscopic identification and enumeration.

### Enumeration and biomass estimation of the photosynthetic community

Lugol’s iodine preserved samples were homogenized briefly (Ultra-Turrax T25 IKA, Germany, 3 × 2 s, 9.5 min^−1^) to break up filaments, but avoid cell damage. The samples were diluted in Milli-Q water and identification and enumeration performed using a light microscope (200× magnification, Nikon Optiphot-2, Japan), and a Malassez chamber (Marienfeld, Germany). All cells contained in 25 squares of the Malassez chamber were counted and analysis of triplicate aliquots undertaken. Cell identification and biovolumes were calculated as outlined previously^52^.

Chl-*a* concentrations were used as a proxy of biofilm biomass (µg of Chl-*a* per cm^2^). Biofilm samples and glass fiber filters with concentrated biomass (for site T1 in June 2013) were placed in Falcon tubes (15 mL) covered with aluminium foil, frozen until analysis, and Chl-*a* was extracted with methanol. Chl-*a* concentrations were estimated according to the protocol described previously by Echenique-Subiabre *et al*.^52^.

### Molecular analysis of biofilm communities

Triplicates subsamples from each site were lyophilized (FreeZone2.5, Labconco, USA) and DNA extracted from ca. 50 mg using a Power Biofilm® DNA Isolation Kit (MOBIO, USA) following the manufacturer’s instructions. Nucleic acid concentrations were determined by spectrophotometry (Nanodrop 1000, Thermo Fischer Inc, USA) and samples stored at −20 °C. A region of the 16S rRNA gene (~394 base pair (bp)) including the variable region V4-V5 was amplified and sequenced using the 515 F and 909 R universal primers^55^ in a commercial facility (Research and Testing Laboratories, Lubbock, TX, USA) using Illumina MiSeq sequencing technology.

### Bioinformatics analysis

The Illumina MiSeq sequencing of the 16S rRNA gene produced 2,085,469 raw sequences. All sequences were processed by the Research and Testing Laboratories (Lubbock, TX, USA), according to their pipeline^56^. Briefly, after denoising (*i*.*e*., removing of short sequences and singletons) and chimera checking, the remaining sequences were clustered using UPARSE pipeline^57^ at 97% similarity threshold. The centroid sequences of each cluster were run against a database of high quality sequences derived from the NCBI database using the USEARCH global alignment algorithm^58^. The number of read per sample ranged from 604 to 57,391. Of the 95 biofilm samples sequenced, three were removed from our analyses because an insufficient number of reads was obtained. Rarefying (randomly down-sampling using software R version 3.1.0^59^) resulted in a total of 820,456 16S rRNA sequences (8,918 sequences per sample), which clustered in 2,198 operational taxonomic units (OTUs). In order to study the BCs associated to *Phormidium* biofilms, a second analysis was performed after removing cyanobacteria and no-hit (CNH) sequences. The number of reads ranged between 32 to 30,560 per sample and a total of 402 sequences per sample were selected for further analysis. Thus, only 82 samples were retained (at least two replicates per sampling site). Rarefying resulted in 32,964 16S rRNA sequences, which clustered in 1,004 OTUs.

OTUs were classified as abundant when they comprised ≥1% of the total sequences, intermediate when between <1% and >0.01%, and rare when ≤0.01% as previously defined by Mangot *et al*.^60^.

### Statistical analysis

The R software (version 3.1.0^59^) was used for statistical analyses of the data. Differences were considered significant when *P* < 0.05. The effect of sampling site and date on cyanobacteria biovolume proportions and abundance of microbial taxa were analysed using two-way ANOVA, followed by multiple comparisons using Tukey’s honestly significant difference (HSD) post-hoc test.

Rarefaction curves were calculated using package ‘vegan’^61^. Hellinger transformations were undertaken on Illumina sequencing data. Beta-diversity was calculated to measure the variation in BC among sites and dates using Bray-Curtis dissimilarities performed at order and OTU levels (package ‘vegan’). Principal component analyses (PCA) were performed on rarefied and transformed data (Hellinger transformation) using the ‘Ade4TkGUI’ package^62^ in order to visualise samples distribution based on their order and OTU structures. Permutational Multivariate ANOVA (PERMANOVA)^63,64^ was performed on Bray-Curtis dissimilarities to test for differences in BC among sites and among sampling times (countries, sites and dates) including factorial and nested designs using package ‘vegan’ and ‘BiodiversityR’^65^ respectively. Finally, the relationship between geographical distance and Bray-Curtis dissimilarity was performed using the package ‘fossil’^66^, longitude and latitude coordinates were transformed to kilometres as measured in a straight line. In order to remove temporal variability (month and year), to analyse exclusively *Phormidium* biofilm samples, and to capture the totality of the sampling sites, only samples from August 2013 were considered for the Tarn River. Therefore, this analysis was performed on biofilms collected at the five sampling sites for the Tarn River, and nine sampling sites from New Zealand rivers (samples from site WMA were excluded because biofilms were dominated by diatoms). Mantel statistic based on Pearson’s product-moment correlation (package ‘vegan’) was performed between dissimilarity matrix and permutation (999) used to evaluate statistical significance.

## Electronic supplementary material

Supplementary Tables and Figures

## Data Availability

The raw sequencing data supporting the results of this article can be found in the European Nucleotide Archive (ENA) under the study accession number PRJEB23673.
