# Supplementary material for: Multiple processes acting from local to large geographical scales shape bacterial communities associated with Phormidium (cyanobacteria) biofilms in French and New Zealand rivers
Source: Sci Rep. 2018 Sep 26;8:14416. doi: 10.1038/s41598-018-32772-w (PMC6158260; doi:10.1038/s41598-018-32772-w)
Supplement: Supplementary file 1 — Supplementary Tables and Figures [file 41598_2018_32772_MOESM1_ESM.docx]

**Supplementary information**

**Multiple processes acting from local to large geographical scales shape bacterial communities associated with *Phormidium* (cyanobacteria) biofilms in French and New Zealand rivers**

Isidora Echenique-Subiabre^1,2,3^, Anouk Zancarini^1^, Mark W. Heath^4,5^, Susanna A. Wood^6,7^, Catherine Quiblier^2,8^, Jean-François Humbert^1*^

^1^ INRA, Université Pierre et Marie Curie, iEES Paris, 4 Place Jussieu, 75252 Paris Cedex, France

^2^ Unité Molécules de Communication et Adaptation des Microorganismes (MCAM, UMR 7245), Muséum National d’Histoire Naturelle, CNRS, Case 39, 57 rue Cuvier 75005 Paris, France

^3^ Present address: GEMA Center for Genomics, Ecology and Environment, Universidad Mayor. Camino La Pirámide 5750, Santiago, Chile.

^4^ School of Biological Sciences, Victoria University of Wellington, PO Box 600, Wellington, New Zealand

^5^ Present address: Greater Wellington Regional Council, Shed 39, 2 Fryatt Quay, Pipitea, Wellington 6111, New Zealand

^6^ Cawthron Institute, Private Bag 2, 7001 Nelson, New Zealand

^7^ Environmental Research Institute, University of Waikato, Private Bag 3105, 3240 Hamilton, New Zealand

^8^ Université Paris Diderot, 5 rue T. Mann, 75013 Paris, France

*Corresponding author: J-F Humbert (jean-francois.humbert@upmc.fr)

**Supplementary Tables**

**Table S1:** Physico-chemical and biological parameters of sites at the Tarn River. Chlorophyll-*a* (Chl-*a*), Non determined (ND). See Fig. S1 for site names and locations.

| **Site** | **Month/Year** | **Flow velocity (m s^-1^)** | **Depth (cm)** | **pH** | **Temperature (°C)** | **Chl-*a* (μg cm^-2^)** | **Cyanobacteria proportion (%)** | **Diatom proportion (%)** | **Green algae proportion (%)** | ***Phormidium* cover (%)** |
| --- | --- | --- | --- | --- | --- | --- | --- | --- | --- | --- |
| T1 | June 2013 | 0.4 ± 0.2 | 27.3 ± 5.9 | 8.1 | 14.9 | 2.1 ± 1.4 | 1 ± 0.4 | 97.7 ± 1.6 | 1.4 ± 1.5 | < 5 |
|  | July 2013 | ND | ND | ND | ND | ND | 84.9 ± 15.3 | 14.5 ± 15 | 0.6 ± 0.4 | <5 |
|  | August 2013 | ND | ND | 7.8 | 22.7 | 23.1 ± 6.4 | 96 ± 2.5 | 3.6 ± 2.7 | 0.3 ± 0.2 | <5 |
|  | September 2013 | 0.6 ± 0.2 | 19.8 ± 2.9 | 7.9 | 18.0 | 19.9 ± 9.5 | 69 ± 6.4 | 30.2 ± 6.2 | 0.8 ± 1.1 | 13.9 ± 7.8 |
|  | June 2014 | 0.6 ± 0.2 | 20.3 ± 13 | 8.8 | 19.8 | 8.9 ± 3.7 | 48 ± 64 | 50.8 ± 63 | 0.8 ± 1.1 | <5 |
|  | July 2014 | ND | ND | ND | 19.3 | 6.2 ± 0.7 | 54.1 ± 15.7 | 41.8 ± 15.6 | 4.1 ± 1.3 | <5 |
|  | August 2014 | ND | ND | 8.1 | 17.0 | 17.7 ± 4.9 | 78.4 ± 2.7 | 21.5 ± 2.7 | 0.1 ± 0.1 | <5 |
|  | September 2014 | ND | ND | ND | 18.6 | 30.2 ± 10.1 | 86.6 ± 6 | 12.5 ± 5 | 0.9 ± 1.1 | <5 |
| T2 | August 2013 | 0.3 ± 0.2 | 38.9 ± 15.2 | 7.9 | 20.7 | 9.3 ± 4.6 | 76.6 ± 27.3 | 22.4 ± 26.3 | 1 ± 1.1 | 34.1 ± 26 |
|  | September 2013 | 0.3 ± 0.2 | 16.1 ± 5.4 | 7.8 | 18.6 | 17.5 ± 7.6 | 45 ± 18.9 | 52 ± 18 | 3 ± 1.2 | 22.2 ± 11.3 |
|  | June 2014 | 0.4 ± 0.2 | 48.3 ± 10.9 | 8.6 | 17.3 | 10.6 ± 4.5 | 84.5 ± 3.6 | 10.8 ± 5.9 | 4.7 ± 2.6 | 24.5 ± 16.1 |
|  | July 2014 | 0.3 ± 0.2 | 47.1 ± 12.6 | 8.6 | 21.4 | 6.4 ± 2.3 | 72.7 ± 9.9 | 24.8 ± 8.3 | 2.5 ± 1.9 | 32.2 ± 25.1 |
|  | August 2014 | 0.3 ± 0.2 | 38.3 ± 22 | 8.1 | 17.5 | 10.3 ± 7.3 | 77.4 ± 14.4 | 22.2 ± 14 | 0.4 ± 0.4 | 22.5 ± 24.6 |
|  | September 2014 | 0.5 ± 0.1 | 54 ± 7.7 | ND | 21.1 | 5.5 ± 2.5 | 60.5 ± 22 | 32.7 ± 22.6 | 6.9 ± 1.9 | 45.5 ± 14.2 |
| T3 | August 2013 | 0.5 ± 0.2 | 27.9 ± 11.9 | 8.1 | 21.1 | 9.4 ± 4 | 83.7 ± 6 | 12.7 ± 7.2 | 3.5 ± 5.2 | 12.9 ± 8.5 |
| T4 | August 2013 | 0.3 ± 0.1 | 31.3 ± 14.5 | 8.0 | 20.2 | 8.6 ± 3.8 | 92 ± 2.6 | 6.2 ± 4.3 | 1.8 ± 1.8 | 31.9 ± 26.8 |
|  | September 2013 | 0.2 ± 0.2 | 57.4 ± 7.2 | 7.9 | 16.3 | ND | 51.9 ± 23.7 | 36.9 ± 18.8 | 11.3 ± 13.3 | 20.7 ± 16.7 |
|  | June 2014 | ND | ND | 8.6 | 16.8 | ND | 75.1 ± 25.9 | 24.2 ± 25.3 | 0.7 ± 0.6 | <5 |
|  | July 2014 | ND | ND | ND | 20.9 | ND | 33.8 ± 37.1 | 58.3 ± 36 | 7.9 ± 4.2 | <5 |
|  | August 2014 | ND | ND | 8.0 | 16.1 | 20.5 ± 9.1 | 97.4 ± 1.6 | 2.5 ± 1.5 | 0 ± 0 | <5 |
|  | September 2014 | ND | ND | ND | 20.0 | 29.5 ± 8.6 | 88.7 ± 9.9 | 10 ± 10 | 1.3 ± 0.6 | <5 |
| T5 | August 2013 | 0.6 ± 0.2 | 35.5 ± 9.9 | 8.1 | 17.9 | 17.7 ± 8.7 | 95.4 ± 1.2 | 4.1 ± 0.8 | 0.5 ± 0.4 | 7.5 ± 9.8 |
|  |  |  |  |  |  |  |  |  |  |  |

**Table S2:** Physico-chemical and biological parameters of different sites from New Zealand Rivers. Chlorophyll-*a* (Chl-*a*), Non determined (ND). See Fig. S1 for site names and locations.

| **Site** | **Flow velocity (m s^-1^)** | **Depth (cm)** | **Chl-a (μg cm^-2^)** | **Cyanobacteria proportion (%)** | **Diatom proportion (%)** | **Green algae proportion (%)** | ***Phormidium* cover (%)** |
| --- | --- | --- | --- | --- | --- | --- | --- |
| K | ND | ND | 11.9 ± 5.2 | 87.1 ± 4.9 | 12.8 ± 4.9 | 0.1 ± 0.1 | 56 ± 20.7 |
| TP | ND | ND | 21.6 ± 3.8 | 90.1 ± 4.8 | 8.2 ± 3.5 | 1.7 ± 1.3 | 51 ± 27.5 |
| TW | ND | ND | 25.6 ± 2.5 | 89.3 ± 3 | 10.6 ± 2.9 | 0.1 ± 0.1 | 17.2 ± 19.6 |
| M | ND | ND | 20.8 ± 5.1 | 84.6 ± 3.8 | 14.3 ± 3.8 | 1.1 ± 0.9 | 16.2 ± 9.2 |
| W | ND | ND | 40.7 ± 8.3 | ND | ND | ND | 11 ± 13.4 |
| WRP | 0.5 ± 0.1 | 15.6 ± 0 | ND | 55.7 ± 21.8 | 44.3 ± 21.9 | 0 ± 0 | 15 ± 6.1 |
| WN | 0.5 ± 0.1 | 16 ± 0 | 20.2 ± 7.6 | 54.1 ± 47.1 | 45.4 ± 46.2 | 0.5 ± 0.9 | <5 |
| WMP | 0.4 ± 0.3 | 18.8 ± 0.1 | ND | ND | ND | ND | <5 |
| MOT | 1.1 ± 0.1 | 28 ± 0 | 19.8 ± 4.6 | 93.2 ± 2.5 | 6.8 ± 2.5 | 0 ± 0 | 14.2 ± 14.5 |
| WMA | 0.5 ± 0.3 | 13.8 ± 0 | 30.7 ± 12.1 | 22.3 ± 16.3 | 55.6 ± 23.4 | 22.1 ± 11.2 | <5 |

**Supplementary Table 3:** Statistical analyses of cyanobacterial proportion of the Tarn (2013 and 2014) and New Zealand rivers. Asterisk denotes interaction between factors. Abbreviations: Aug, August; Sept, September. See Fig. S1 for site names and locations.

|  | Tarn 2013 | | Tarn 2014 | | New Zealand 2013 | |
| --- | --- | --- | --- | --- | --- | --- |
| Variable | Two-way ANOVA test (*P*) | Tukey test (*P*) | Two-way ANOVA test (*P*) | Tukey test (*P*) | ANOVA test (*P*) | Tukey test (*P*) |
| Cyanobacterial proportion (%) | Site (0.009)  Month (<0.001)  Site*Month (0.72) | T1-T5 (0.014)  T2-T5 (0.018)  June-July (<0.001)  June-Aug (<0.001)  June-Sept (<0.001) | Site (0.93)  Month (0.04)  Site*Month (0.17) | July-Aug (0.035) | Site (0.003) | WMA-K (0.015)  WMA-TP (0.01)  WMA-TW (0.01)  WMA-M (0.02)  WMA-MOT (0.007) |
|  |  | July-Sept (0.01)  Aug-Sept (0.004) |  |  |  |  |

**Supplementary Table 4:** Taxonomic composition of the bacterial community of the biofilms (excluding cyanobacteria and no-hit) from this study based on the normalized 16S rRNA gene sequences characterized by Illumina sequencing approach. Operational taxonomic units (OTUs) were classified as abundant when they comprised ≥1% of the total sequences, intermediate (Interm.) when between <1% and >0.01%, and rare when ≤0.01%.

| **Taxonomic Affiliation** | | | **Reads** | **Total** | **Number of OTUs at the 97% cut-off** | | | |
| --- | --- | --- | --- | --- | --- | --- | --- | --- |
| **Phylum** | **Class** | **Order** | **(%)** | **sequences number** | **Total** | **Abundant** | **Interm.** | **Rare** |
| Acidobacteria | Acidobacteriia | Acidobacteriales | 0.1 | 37 | 11 |  | 2 | 9 |
|  |  | Unclassified | 0.0 | 9 | 3 |  | 1 | 2 |
|  | Holophagae | Holophagales | 0.1 | 23 | 3 |  | 1 | 2 |
| Actinobacteria | Actinobacteria | Acidomicrobiales | 0.0 | 4 | 3 |  |  | 3 |
|  |  | Actinomycetales | 0.5 | 167 | 15 |  | 8 | 7 |
|  |  | Solirubrobacterales | 0.0 | 8 | 2 |  | 1 | 1 |
|  |  | Unclassified | 0.1 | 49 | 6 |  | 2 | 4 |
|  | Unclassified |  | 0.0 | 9 | 4 |  |  | 4 |
| Aquificae | Unclassified |  | 0.0 | 1 | 1 |  |  | 1 |
| Armatimonadetes | Armatimonadia | Armatimonadales | 0.0 | 13 | 1 |  | 1 |  |
| Bacteroidetes | Bacteroidia | Bacteroidales | 0.3 | 85 | 4 |  | 2 | 2 |
|  | Cytophagia | Cytophagales | 4.9 | 1621 | 75 | 1 | 29 | 45 |
|  | Flavobacteriia | Flavobacteriales | 8.9 | 2920 | 45 | 2 | 29 | 14 |
|  |  | Unclassified | 0.3 | 115 | 4 |  | 1 | 3 |
|  | Sphingobacteriia | Sphingobacteriales | 6.8 | 2246 | 67 | 2 | 30 | 35 |
|  |  | Unclassified | 1.0 | 343 | 17 |  | 12 | 5 |
|  | Unclassified |  | 1.4 | 460 | 36 |  | 19 | 17 |
| Chloroflexi | Anaerolineae | Anaerolineales | 0.0 | 9 | 1 |  | 1 |  |
|  | Caldilineae | Caldilineales | 0.0 | 2 | 2 |  |  | 2 |
|  | Chloroflexia | Herpetosiphonales | 0.0 | 1 | 1 |  |  | 1 |
|  | Unclassified |  | 0.0 | 8 | 4 |  |  | 4 |
| Deferribacteres | Deferribacteres | Unclassified | 0.0 | 11 | 1 |  | 1 |  |
| Deinococcus-Thermus | Deinococci | Deinococcales | 0.4 | 124 | 3 |  | 3 |  |
| Fibrobacteres | Fibrobacteria | Fibrobacterales | 0.0 | 3 | 1 |  |  | 1 |
| Firmicutes | Bacilli | Bacillales | 0.1 | 23 | 2 |  | 1 | 1 |
|  |  | Lactobacillales | 0.0 | 4 | 2 |  |  | 2 |
|  | Clostridia | Clostridiales | 0.1 | 22 | 7 |  | 2 | 5 |
|  |  | Unclassified | 0.0 | 5 | 3 |  |  | 3 |
| Fusobacteria | Fusobacteriia | Fusobacteriales | 0.0 | 10 | 1 |  | 1 |  |
| Gemmatimonadetes | Gemmatimonadetes | Gemmatimonadales | 0.1 | 40 | 2 |  | 1 | 1 |
| Planctomycetes | Phycisphaerae | Phycisphaerales | 0.0 | 5 | 1 |  | 1 |  |
|  | Planctomycetia | Planctomycetales | 1.0 | 328 | 60 |  | 17 | 43 |
|  |  | Unclassified | 0.0 | 13 | 7 |  |  | 7 |
|  | Unclassified |  | 0.1 | 28 | 11 |  | 2 | 9 |
| Proteobacteria | Alphaproteobacteria | Caulobacterales | 0.7 | 228 | 10 |  | 8 | 2 |
|  |  | Rhizobiales | 2.9 | 963 | 54 |  | 31 | 23 |
|  |  | Rhodobacterales | 10.8 | 3551 | 30 | 2 | 12 | 16 |
|  |  | Rhodospirillales | 1.9 | 616 | 23 |  | 7 | 16 |
|  |  | Rickettsiales | 0.1 | 31 | 13 |  | 2 | 11 |
|  |  | Sphingomonadales | 8.0 | 2630 | 65 | 1 | 33 | 31 |
|  |  | Unclassified | 1.9 | 619 | 10 | 1 | 1 | 8 |
|  | Betaproteobacteria | Burkholderiales | 12.2 | 4011 | 43 | 5 | 22 | 16 |
|  |  | Methylophilales | 0.4 | 140 | 4 |  | 1 | 3 |
|  |  | Neisseriales | 0.2 | 55 | 6 |  | 3 | 3 |
|  |  | Nitrosomonadales | 0.0 | 1 | 1 |  |  | 1 |
|  |  | Procabacteriales | 0.0 | 2 | 1 |  |  | 1 |
|  |  | Rhodocyclales | 2.2 | 718 | 15 |  | 8 | 7 |
|  |  | Unclassified | 6.5 | 2144 | 31 | 2 | 15 | 14 |
|  | Deltaproteobacteria | Bdellovibrionales | 0.0 | 10 | 5 |  |  | 5 |
|  |  | Desulfobacterales | 0.0 | 7 | 1 |  |  | 1 |
|  |  | Desulfovibrionales | 0.0 | 1 | 1 |  |  | 1 |
|  |  | Desulfuromonadales | 0.0 | 11 | 5 |  |  | 5 |
|  |  | Myxococcales | 0.2 | 73 | 12 |  | 6 | 6 |
|  |  | Syntrophobacterales | 0.0 | 1 | 1 |  |  | 1 |
|  |  | Unclassified | 0.3 | 111 | 15 |  | 7 | 8 |
|  | Gammaproteobacteria | Acidithiobacillales | 0.0 | 8 | 1 |  | 1 |  |
|  |  | Aeromonadales | 1.6 | 541 | 3 | 1 | 1 | 1 |
|  |  | Chromatiales | 0.1 | 22 | 3 |  | 2 | 1 |
|  |  | Enterobacteriales | 0.1 | 24 | 1 |  | 1 |  |
|  |  | Legionalles | 0.1 | 18 | 9 |  | 1 | 8 |
|  |  | Methylococcales | 0.0 | 12 | 4 |  | 1 | 3 |
|  |  | Oceanospirillales | 0.0 | 1 | 1 |  |  | 1 |
|  |  | Pseudomonadales | 0.4 | 133 | 10 |  | 6 | 4 |
|  |  | Thiotrichales | 0.0 | 7 | 2 |  |  | 2 |
|  |  | Xanthomonadales | 6.4 | 2118 | 35 | 2 | 18 | 15 |
|  |  | Unclassified | 0.1 | 39 | 3 |  | 1 | 2 |
|  | Oligoflexia | Oligoflexales | 0.0 | 2 | 1 |  |  | 1 |
|  | Unclassified |  | 12.2 | 4034 | 73 | 3 | 30 | 40 |
| Spirochaetes | Spirochaetia | Spirochaetales | 0.0 | 5 | 2 |  |  | 2 |
| Verrucomicrobia | Opitutae | Opitutales | 0.1 | 39 | 8 |  | 1 | 7 |
|  | Verrucomicrobiae | Verrucomicrobiales | 0.1 | 29 | 6 |  | 1 | 5 |
|  | Unclassified |  | 0.1 | 24 | 3 |  | 1 | 2 |
| Unclassified |  |  | 3.8 | 1239 | 87 |  | 32 | 55 |
| Total |  |  |  | 32964 | 1004 | 22 | 421 | 561 |

**Supplementary Table 5:** Beta-diversity at different sampling scales (excluding cyanobacterial and no-hit). Bray-Curtis dissimilarity was calculated as average from relative abundance data at order (≥1% most abundant) and operational taxonomic unit (OTU) level, Hellinger transformations were performed at both levels.

| Sampling Campaign | Comparison | Beta-diversity | |
| --- | --- | --- | --- |
|  |  | Order level | OTU level |
|  |  | Bray-Curtis | Bray-Curtis |
| Tarn 2014 | All | 0.16 | 0.48 |
|  | Intra-site (T1) | 0.19 | 0.49 |
|  | Intra-site (T2) | 0.13 | 0.44 |
|  | Intra-site (T4) | 0.18 | 0.51 |
| Tarn 2013 & 2014 | All | 0.18 | 0.51 |
| New Zealand 2013 | All | 0.20 | 0.57 |
|  | Nord Island | 0.20 | 0.61 |
|  | South Island | 0.12 | 0.45 |
|  | Intra-river (Tukituki) | 0.08 | 0.33 |
|  | Intra-river (Wakapuaka) | 0.11 | 0.36 |
| Tarn & New Zealand | All | 0.20 | 0.55 |
|  | Tarn 2013 & New Zealand 2013 | 0.22 | 0.60 |
|  | Tarn 2014 & New Zealand 2013 | 0.19 | 0.60 |
|  | Tarn Aug 2013 & New Zealand | 0.22 | 0.60 |

**Supplementary Table 6:** Results from ANOVA and Tukey test performed on relative abundances of bacterial orders (≥1% most abundant, excluding cyanobacteria and no-hit). Values in bold represent *P* < 0.05. Abbreviations: Aug, August; Sept, September.

| **Sampling campaign** | **Two-way ANOVA test (*P*)** | | | | **Tukey test (*P*)** | | | | | | | | | | |  | |  | |
| --- | --- | --- | --- | --- | --- | --- | --- | --- | --- | --- | --- | --- | --- | --- | --- | --- | --- | --- | --- |
| **Site T1** |  |  | |  |  | |  |  |  | |  | |  | |  |  | |  | |
| **Bacteria order** | **Pyear** | **Pdate** | | **Pdateyear** | **Pyear** | | | | **Pdate** | | | | | | |  | |  | |
| Rhizobiales | 0.058 | **0.000** | | **0.000** |  | |  |  | June (25.4) a | | July (6) b | | | Sept (5) b | Aug (4.4) b |  | |  | |
| Rhodobacterales | 0.286 | **0.001** | | **0.028** |  | |  |  | June (60.2) a | | July (31.8) b | | | Sept (24.5) b | Aug (20.4) b |  | |  | |
| Rhodospirillales | 0.966 | 0.526 | | 0.347 |  | |  |  |  | |  | | |  |  |  | |  | |
| Sphingomonadales | 0.133 | **0.001** | | 0.209 |  | |  |  | June (81) a | | July (23.2) b | | | Aug (15.8) b | Sept (15.67) b |  | |  | |
| Cytophagales | **0.028** | **0.032** | | 0.146 | 2013 (24.5) a | | 2014 (11.45) b |  | Aug (29.4) a | | Sept (20.83) ab | | | July (13.4) ab | June (6.4) b |  | |  | |
| Flavobacteriales | **0.019** | **0.001** | | **0.007** | 2014 (58.82) a | | 2013 (31.5) b |  | Sept (81) a | | Aug (58.4) ab | | | July (35.4) bc | June (1.4) c |  | |  | |
| Sphingobacteriales | 0.972 | **0.001** | | **0.004** |  | |  |  | Sept (32.5) a | | July (23) a | | | Aug (19.8) ab | June (4.6) b |  | |  | |
| Burkholderiales | 0.060 | 0.368 | | 0.212 |  | |  |  |  | |  | | |  |  |  | |  | |
| Rhodocyclales | **0.000** | 0.340 | | 0.360 | 2014 (6) a | | 2013 (1.6) b |  |  | |  | | |  |  |  | |  | |
| Aeromonadales | 0.226 | **0.017** | | 0.282 |  | |  |  | Aug (9.2) a | | Sept (8.67) a | | | July (7.4) ab | June (0.4) b |  | |  | |
| Xanthomonadales | 0.478 | **0.000** | | **0.001** |  | |  |  | Aug (43.4) a | | July (20.8) b | | | Sept (15.67) b | June (8.2) b |  | |  | |
| Planctomycetales | 0.071 | 0.461 | | 0.104 |  | |  |  |  | |  | | |  |  |  | |  | |
| **Tarn 2014** |  |  | |  |  | |  |  |  | |  | | |  |  |  | |  | |
| **Bacteria order** | **Psite** | **Pdate** | | **Psitedate** | **Psite** | | | | **Pdate** | | | | | | |  | |  | |
| Rhizobiales | **0.034** | **0.000** | | **0.000** | T1 (12) a | | T2 (10.09) ab | T4 (6) b | June (18.42) a | | July (10.25) b | | Aug (7.25) b | | Sept (3.78) b |  | |  | |
| Rhodobacterales | 0.546 | **0.022** | | **0.001** |  | |  |  | June (50) a | | July (49.38) a | | Aug (34.75) ab | | Sept (26.67) b |  | |  | |
| Rhodospirillales | 0.833 | 0.233 | | 0.264 |  | |  |  |  | |  | |  | |  |  | |  | |
| Sphingomonadales | 0.228 | **0.038** | | 0.104 |  | |  |  | June (39.71) a | | Aug (27.88) ab | | Sept (25.44) ab | | July (20.25) b |  | |  | |
| Cytophagales | **0.016** | 0.050 | | **0.046** | T4 (25.8) a | | T2 (13.45) b | T1 (11.45) b |  | |  | |  | |  |  | |  | |
| Flavobacteriales | **0.039** | **0.000** | | **0.005** | T1 (58.82) a | | T2 (46.45) ab | T4 (31) b | Sept (77.89) a | | Aug (45.88) b | | June (28) b | | July (25.5) b |  | |  | |
| Sphingobacteriales | 0.500 | 0.071 | | 0.096 |  | |  |  |  | |  | |  | |  |  | |  | |
| Burkholderiales | 0.733 | **0.041** | | 0.352 |  | |  |  |  | |  | |  | |  |  | |  | |
| Rhodocyclales | **0.007** | 0.937 | | 0.295 | T2 (12.18) a | | T4 (6.3) b | T1 (6) b |  | |  | |  | |  |  | |  | |
| Aeromonadales | **0.013** | 0.073 | | 0.077 | T4 (11.8) a | | T1 (5.36) b | T2 (3.9) b |  | |  | |  | |  |  | |  | |
| Xanthomonadales | 0.206 | 0.584 | | **0.015** |  | |  |  |  | |  | |  | |  |  | |  | |
| Planctomycetales | 0.546 | 0.825 | | 0.331 |  | |  |  |  | |  | |  | |  |  | |  | |
|  | **Two-way ANOVA test (*P*)** | | | **Tukey test (*P*)** | | | | | | | | | | | | | | | |
| **New Zealand 2013** |  | |  |  |  | |  |  |  | |  | |  | |  | |  |  | |
| **Bacteria order** | **Psite** | | | **Psite** | | | | | | | | | | | | | | | |
| Rhizobiales | **0.000** | | | M (25.67) a | | K (23) a | W (16) ab | TW (14.67) ab | | WN (8) ab | | WRP (8) ab | TP (4) b | | WMP (3) b | | WMA (2) b | | MOT (1.5) b |
| Rhodobacterales | 0.406 | | |  | |  |  |  | |  | |  |  | |  | |  | |  |
| Rhodospirillales | **0.012** | | | TP (24.67) a | | TW (18) ab | WMA (17) ab | MOT (15.5) ab | | WN (13.5) ab | | M (11.3) ab | WMP (6.3) ab | | W (4.3) b | | WRP (3.5) b | | K (3.3) b |
| Sphingomonadales | **0.000** | | | W (108) a | | M (84) ab | TP (54.3) abc | TW (40.67) bc | | WRP (30.5) bc | | WN (18) bc | WMA (16) c | | WMP (15.67) c | | K (14) c | | MOT (12) c |
| Cytophagales | **0.000** | | | MOT (49) a | | WN (30.5) ab | WRP (25.5) abc | M (14.67) bc | | WMP (14.67) bc | | TW (12) bc | TP (9.67) bc | | WMA (8.3) bc | | W (8) bc | | K (3.3) c |
| Flavobacteriales | 0.608 | | |  | |  |  |  | |  | |  |  | |  | |  | |  |
| Sphingobacteriales | 0.063 | | |  | |  |  |  | |  | |  |  | |  | |  | |  |
| Burkholderiales | **0.000** | | | WMP (75.3) a | | MOT (75) a | WMA (75) a | TW (51) ab | | WN (43.5) abc | | K (39) abc | TP (26) bc | | WRP (23) bc | | W (10.3) bc | | M (4.67) c |
| Rhodocyclales | 0.477 | | |  | |  |  |  | |  | |  |  | |  | |  | |  |
| Aeromonadales | 0.854 | | |  | |  |  |  | |  | |  |  | |  | |  | |  |
| Xanthomonadales | **0.018** | | |  | |  |  |  | |  | |  |  | |  | |  | |  |
| Planctomycetales | 0.230 | | |  | |  |  |  | |  | |  |  | |  | |  | |  |

**Supplementary Table 7**: First 50 most abundant bacterial operational taxonomic units from this study (excluding cyanobacteria and no-hit).

| **Taxonomic Affiliation** | | | | | **Reads** | **Total** |
| --- | --- | --- | --- | --- | --- | --- |
| **Phylum** | **Class** | **Order** | **Family** | **Genus** | **(%)** | **sequences number** |
| Proteobacteria | Alphaproteobacteria | Rhodobacterales | Rhodobacteraceae | *Rhodobacter* | 6.3 | 2093 |
| Proteobacteria | Unclassified |  |  |  | 4.6 | 1517 |
| Proteobacteria | Alphaproteobacteria | Sphingomonadales | Unclassified |  | 3.3 | 1075 |
| Bacteroidetes | Sphingobacteriia | Sphingobacteriales | Sphingobacteriaceae | *Pedobacter* | 3.2 | 1046 |
| Proteobacteria | Betaproteobacteria | Burkholderiales | Unclassified |  | 3.2 | 1039 |
| Proteobacteria | Gammaproteobacteria | Xanthomonadales | Xanthomonadaceae | *Silanimonas* | 2.9 | 955 |
| Proteobacteria | Betaproteobacteria | Unclassified |  |  | 2.9 | 952 |
| Bacteroidetes | Flavobacteriia | Flavobacteriales | Unclassified |  | 2.7 | 893 |
| Bacteroidetes | Flavobacteriia | Flavobacteriales | Flavobacteriaceae | *Flavobacterium* | 2.3 | 745 |
| Proteobacteria | Unclassified | Unclassified |  |  | 2.2 | 728 |
| Proteobacteria | Alphaproteobacteria | Rhodobacterales | Rhodobacteraceae | *Rhodobacter* | 2.2 | 726 |
| Proteobacteria | Betaproteobacteria | Burkholderiales | Unclassified |  | 2.2 | 712 |
| Proteobacteria | Alphaproteobacteria | Unclassified |  |  | 1.8 | 599 |
| Proteobacteria | Betaproteobacteria | Burkholderiales | Comamonadaceae | *Hydrogenophaga* | 1.8 | 599 |
| Bacteroidetes | Cytophagia | Cytophagales | Cytophagaceae | *Runella* | 1.8 | 578 |
| Proteobacteria | Betaproteobacteria | Unclassified |  |  | 1.7 | 575 |
| Proteobacteria | Betaproteobacteria | Burkholderiales | Comamonadaceae | *Hydrogenophaga* | 1.5 | 493 |
| Proteobacteria | Gammaproteobacteria | Aeromonadales | Aeromonadaceae | *Tolumonas* | 1.4 | 467 |
| Proteobacteria | Unclassified |  |  |  | 1.4 | 463 |
| Proteobacteria | Betaproteobacteria | Burkholderiales | Unclassified |  | 1.3 | 426 |
| Bacteroidetes | Sphingobacteriia | Sphingobacteriales | Sphingobacteriaceae | *Sphingobacterium* | 1.1 | 352 |
| Proteobacteria | Gammaproteobacteria | Xanthomonadales | Unclassified |  | 1.0 | 344 |
| Unclassified Bacteria |  |  |  |  | 0.9 | 283 |
| Bacteroidetes | Cytophagia | Cytophagales | Unclassified |  | 0.8 | 259 |
| Proteobacteria | Betaproteobacteria | Rhodocyclales | Rhodocyclaceae | *Azonexus* | 0.8 | 253 |
| Proteobacteria | Betaproteobacteria | Rhodocyclales | Rhodocyclaceae | *Zoogloea* | 0.8 | 253 |
| Proteobacteria | Unclassified |  |  |  | 0.7 | 232 |
| Proteobacteria | Betaproteobacteria | Unclassified |  |  | 0.7 | 230 |
| Proteobacteria | Gammaproteobacteria | Xanthomonadales | Xanthomonadaceae | *Lysobacter* | 0.6 | 212 |
| Proteobacteria | Unclassified |  |  |  | 0.6 | 210 |
| Proteobacteria | Alphaproteobacteria | Sphingomonadales | Sphingomonadaceae | *Sphingomonas* | 0.6 | 199 |
| Proteobacteria | Alphaproteobacteria | Sphingomonadales | Sphingomonadaceae | *Sphingomonas* | 0.6 | 198 |
| Proteobacteria | Alphaproteobacteria | Rhodospirillales | Acetobacteraceae | *Roseococcus* | 0.6 | 195 |
| Unclassified Bacteria |  |  |  |  | 0.6 | 188 |
| Proteobacteria | Unclassified |  |  |  | 0.6 | 187 |
| Bacteroidetes | Sphingobacteriia | Sphingobacteriales | Sphingobacteriaceae | *Pedobacter* | 0.5 | 173 |
| Proteobacteria | Alphaproteobacteria | Sphingomonadales | Unclassified |  | 0.5 | 169 |
| Bacteroidetes | Sphingobacteriia | Sphingobacteriales | Sphingobacteriaceae | *Sphingobacterium* | 0.5 | 157 |
| Bacteroidetes | Cytophagia | Cytophagales | Cytophagaceae | *Arcicella* | 0.5 | 155 |
| Proteobacteria | Gammaproteobacteria | Xanthomonadales | Xanthomonadaceae | *Lysobacter* | 0.5 | 151 |
| Proteobacteria | Betaproteobacteria | Burkholderiales | Comamonadaceae | *Variovorax* | 0.4 | 145 |
| Bacteroidetes | Flavobacteriia | Flavobacteriales | Unclassified |  | 0.4 | 144 |
| Proteobacteria | Alphaproteobacteria | Rhodobacterales | Rhodobacteraceae | *Rhodobacter* | 0.4 | 143 |
| Proteobacteria | Alphaproteobacteria | Rhodospirillales | Unclassified |  | 0.4 | 136 |
| Proteobacteria | Betaproteobacteria | Methylophilales | Unclassified |  | 0.4 | 135 |
| Bacteroidetes | Flavobacteriia | Flavobacteriales | Flavobacteriaceae | *Flavobacterium* | 0.4 | 125 |
| Proteobacteria | Alphaproteobacteria | Rhizobiales | Rhizobiaceae | *Agrobacterium* | 0.4 | 125 |
| Proteobacteria | Alphaproteobacteria | Rhizobiales | Hyphomicrobiaceae | *Hyphomicrobium* | 0.4 | 124 |
| Proteobacteria | Betaproteobacteria | Unclassified |  |  | 0.4 | 122 |
| Bacteroidetes | Cytophagia | Cytophagales | Cytophagaceae | *Spirosoma* | 0.4 | 120 |
|  |  |  |  |  |  |  |
| Total |  |  |  |  | 68 | 22400 |

**Supplementary Figure Legends**

**Supplementary Figure 1:** Flow rates of Tarn River in 2013 (A) and 2014 (B) measured at Bedoues (doted line) located 28 km upstream and Mostuejouls (solid line) located 38 km downstream from Sainte-Enimie respectively. Arrows show sampling days.


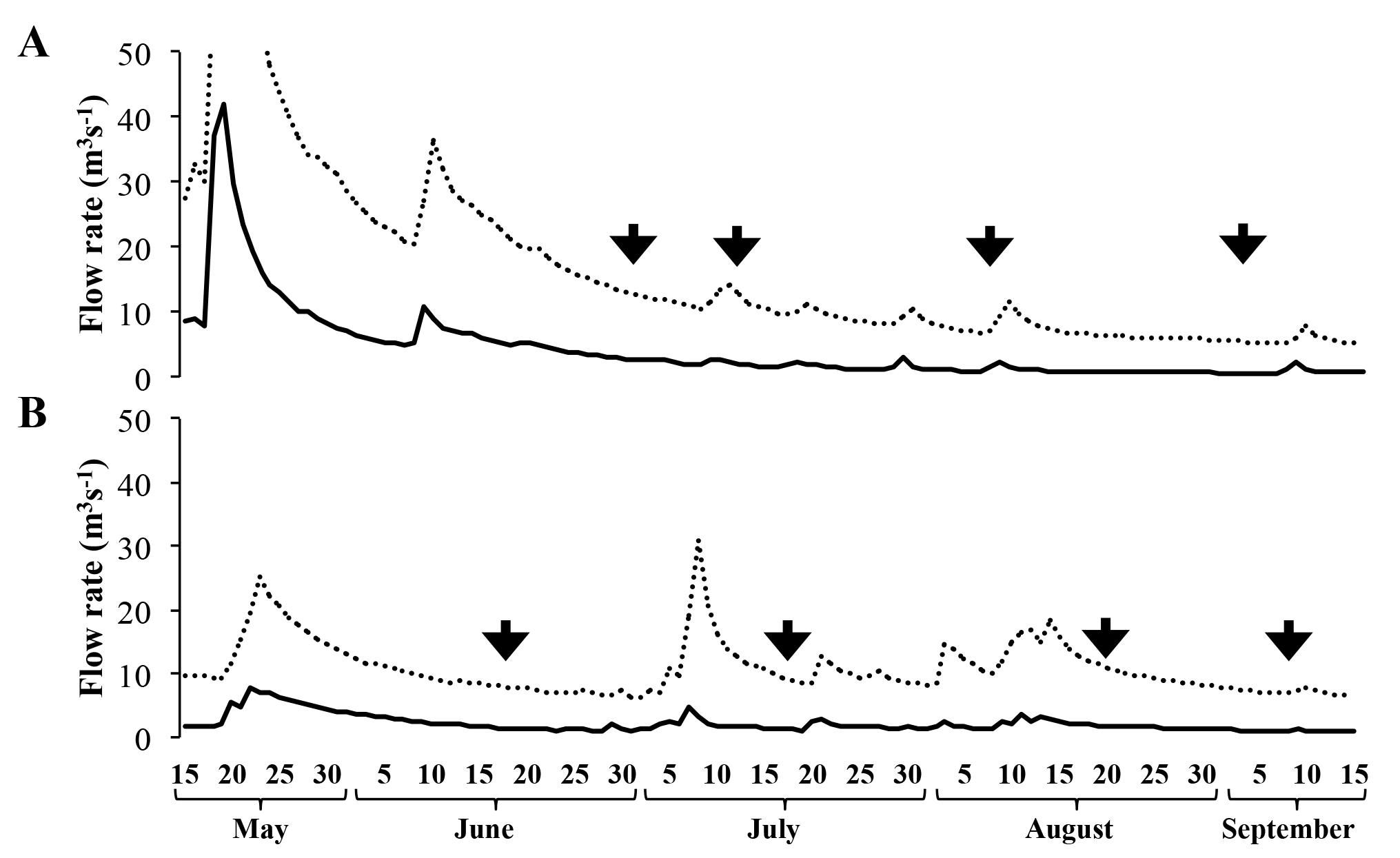


**Supplementary Figure 2: Relative sequence abundance of the dominant bacterial phyla/order composing cyanobacterial biofilms.** Average relative abundances based on 16S rRNA gene fragment sequences are represented for the two years of sampling, in the Tarn River and in New Zealand (NZ) rivers. On the left all phyla are included and on the right cyanobacteria phylum and no-hit are excluded. At the bottom, cluster analysis of the microbial diversity at the order level.


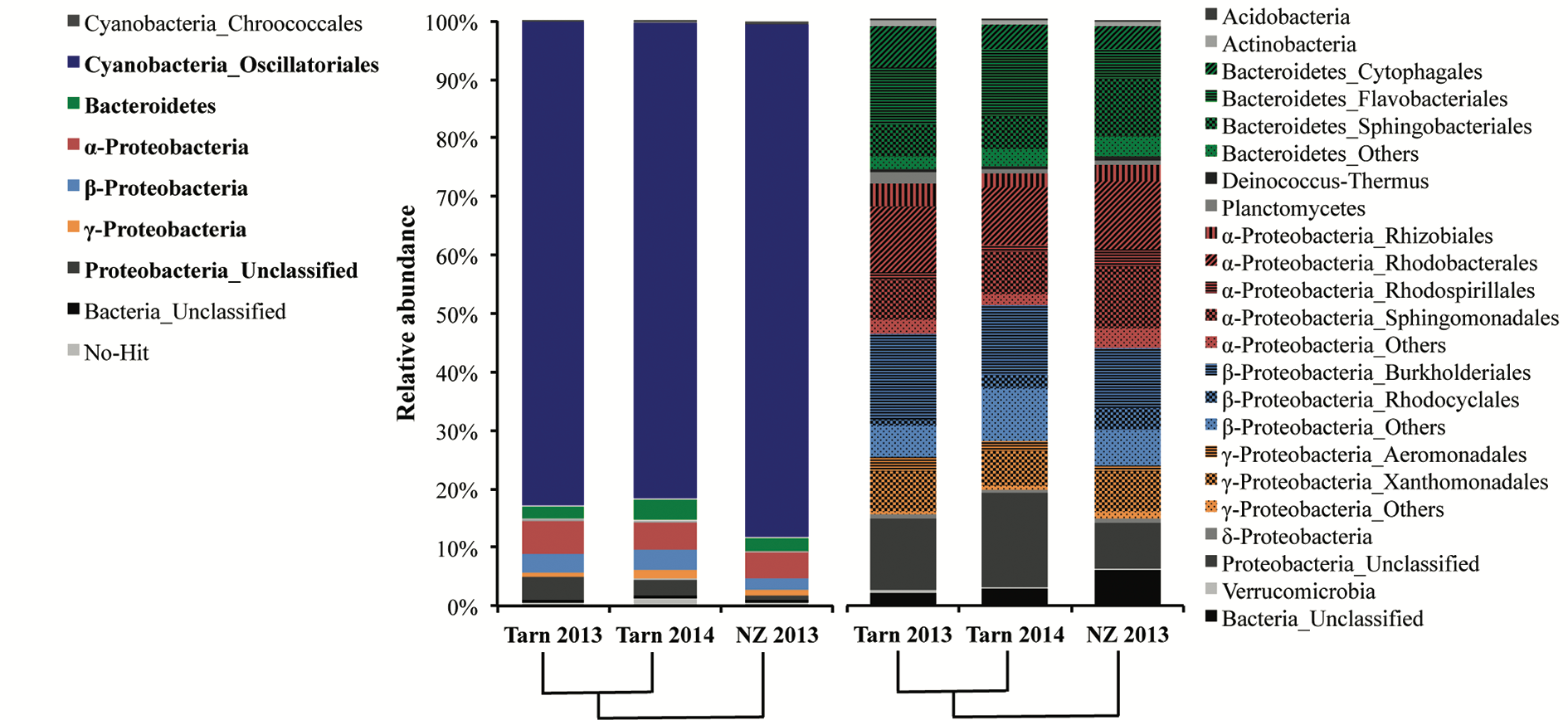


**Supplementary Figure 3:** Principal Component Analysis (PCA) of bacterial orders (most abundant; ≥1%) (A) and operational taxonomic units (OTUs) (B) relative abundances based on 16S rRNA gene fragment (excluding cyanobacteria and no-hit) of biofilm samples from all sampling campaigns (Tarn 2013, 2014 and New Zealand (NZ)). Histograms on the bottom-left part of the graph represent the percentages of variation explained by each principal component. The two components selected for the two dimensional representation are highlighted in black.


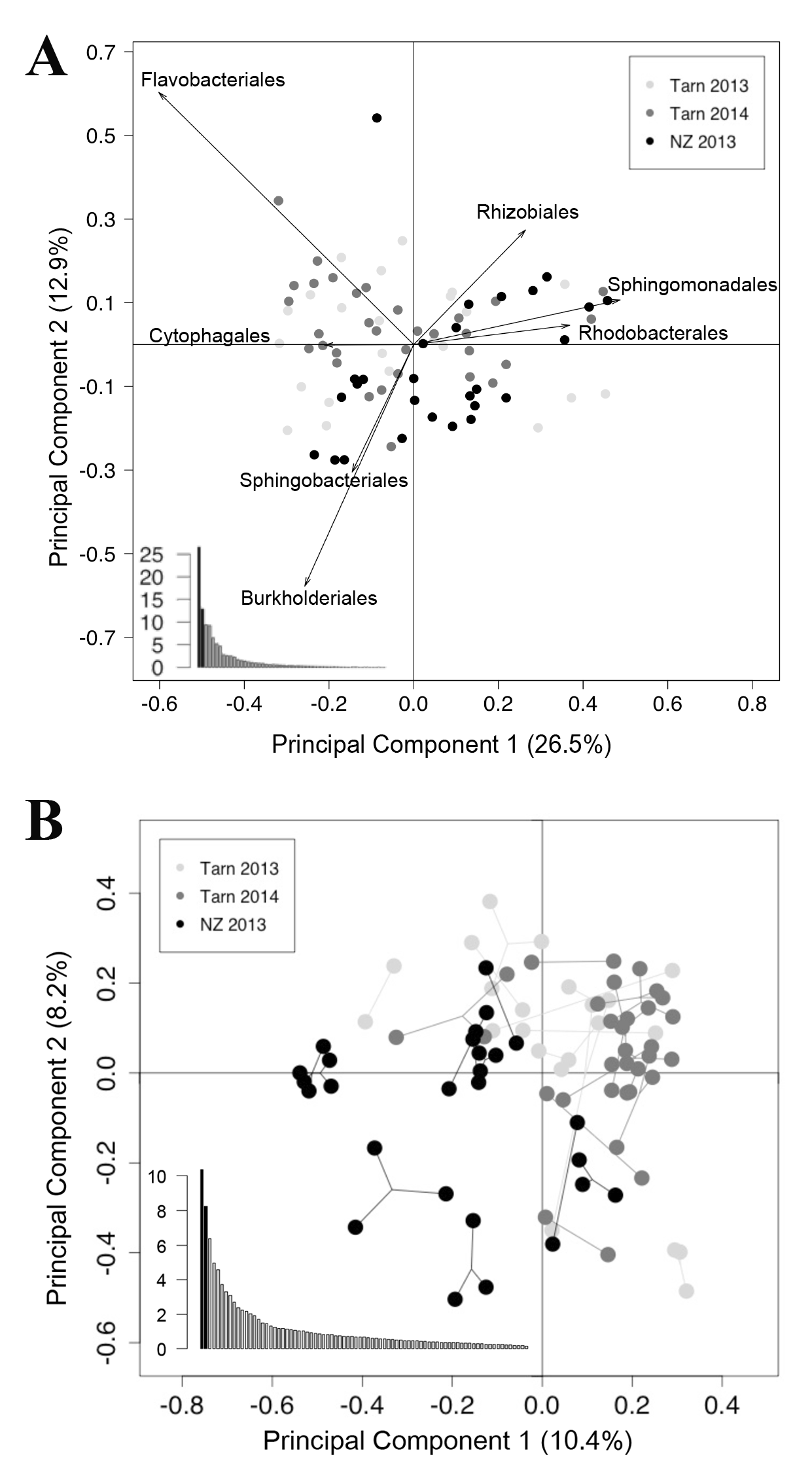


**Supplementary Figure 4:** Rarefaction curves of Tarn and New Zealand (NZ) rivers for total 16S rRNA gene sequences (A-C), and excluding cyanobacteria and no-hit (D-F). OTU = operational taxonomic unit.





**Supplementary Figure 5: Venn diagram of operational taxonomic units (OTUs) of biofilms from the Tarn and New Zealand (NZ) rivers.** OTUs are based on 16S rRNA gene fragment sequences. All OTUs (A) and excluding cyanobacteria and no-hit (B). Abbreviations refer to the median (m_d_) and mean (m_n_) of the number of sequences per OTU.


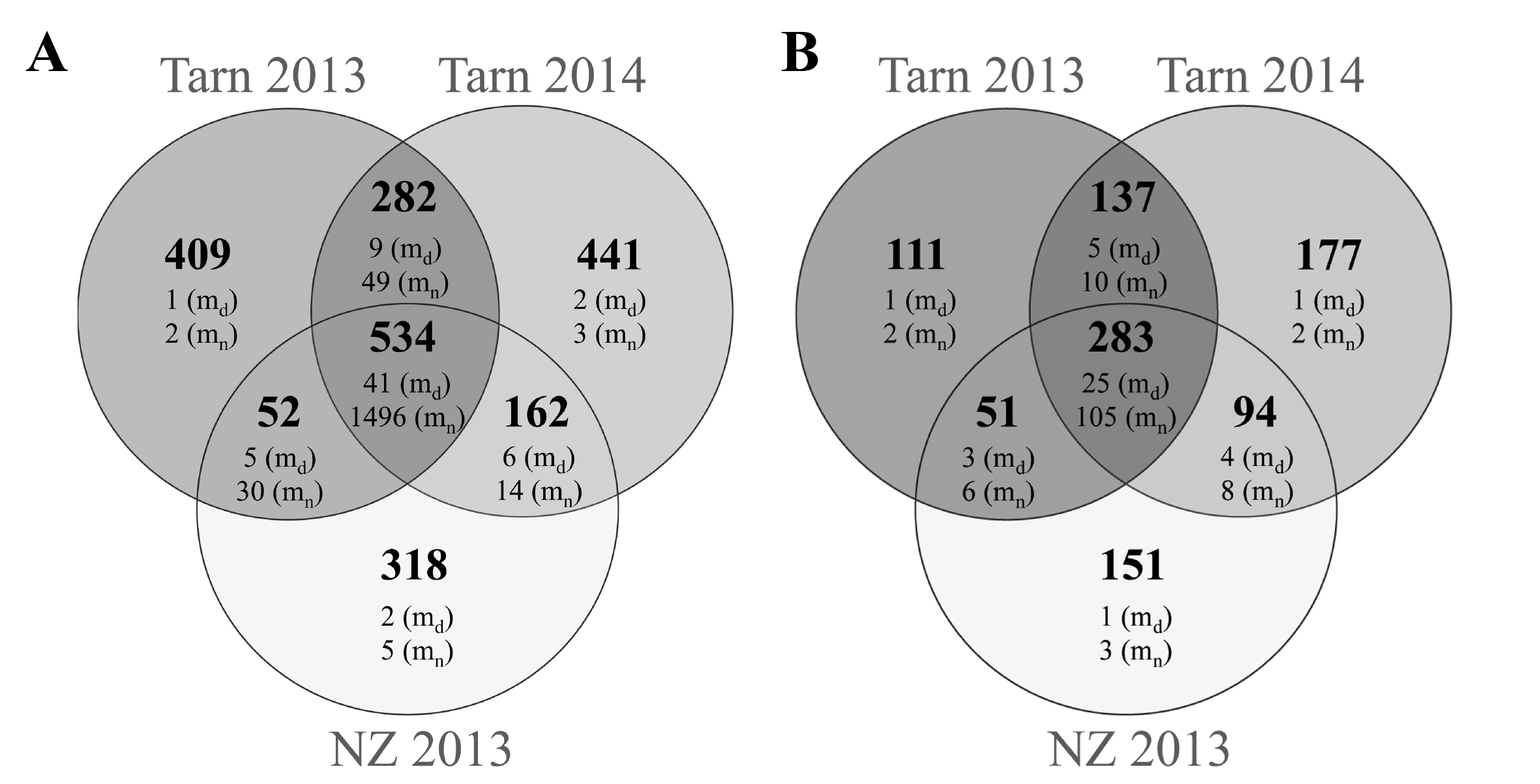


**Supplementary Figure 6:** Bacterial operational taxonomic units occurrence (based on 16S rRNA gene fragment) and sequence number per biofilm sample excluding cyanobacteria and no-hit OTU sequences.





**Supplementary Figure 7:** Geographic distance and Jaccard dissimilarity relationship (based on presence/absence data) in Tarn River (August 2013; open circles on the left), New Zealand (NZ) rivers (black circles in the middle) and between Tarn and New Zealand rivers (open circles at the right) of bacterial 16S rRNA operational taxonomic unit abundances of pooled replicates excluding cyanobacteria and no-hit. Mantel statistics are based on Pearson's product-moment correlation.

**
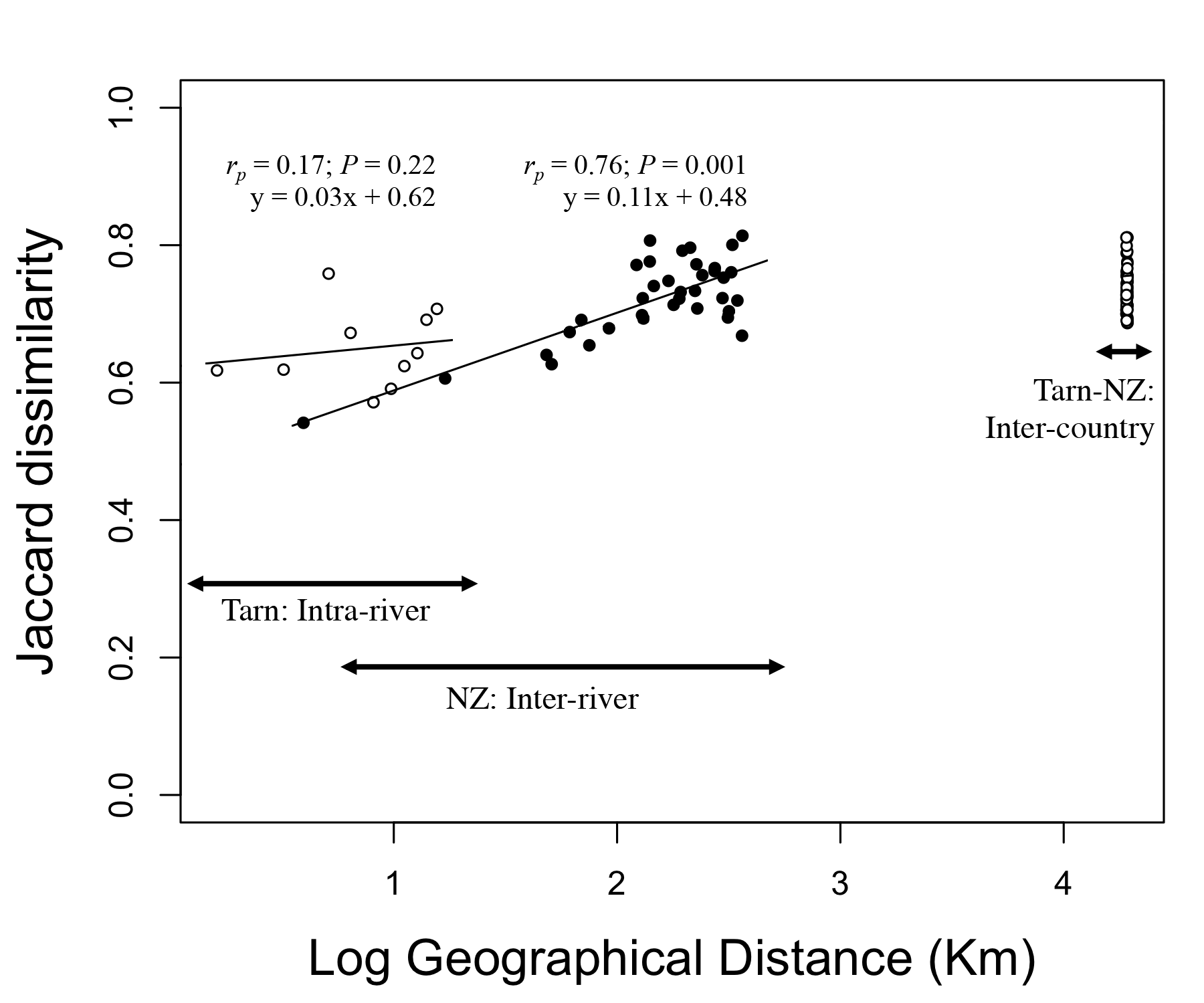
**
